# Thorough Investigation of a Canine Autoinflammatory Disease (AID) Confirms One Main Risk Locus and Suggests a Modifier Locus for Amyloidosis

**DOI:** 10.1371/journal.pone.0075242

**Published:** 2013-10-09

**Authors:** Mia Olsson, Linda Tintle, Marcin Kierczak, Michele Perloski, Noriko Tonomura, Andrew Lundquist, Eva Murén, Max Fels, Katarina Tengvall, Gerli Pielberg, Caroline Dufaure de Citres, Laetitia Dorso, Jérôme Abadie, Jeanette Hanson, Anne Thomas, Peter Leegwater, Åke Hedhammar, Kerstin Lindblad-Toh, Jennifer R. S. Meadows

**Affiliations:** 1 Science for Life Laboratory, Department of Medical Biochemistry and Microbiology, Uppsala University, Uppsala, Sweden; 2 Wurtsboro Veterinary Clinic, Wurtsboro, New York, United States of America; 3 Computational Genetics Section, Department of Clinical Sciences, Swedish University of Agricultural Sciences (SLU), Uppsala, Sweden; 4 Broad Institute of Harvard and Massachusetts Institute of Technology (MIT), Cambridge, MA, United States of America; 5 Department of Clinical Sciences, Cummings School of Veterinary Medicine at Tufts University, North Grafton, Massachusetts, United States of America; 6 Albert Einstein College of Medicine, Bronx, New York, United States of America; 7 ANTAGENE Animal Genetics Laboratory, La Tour de Salvagny (69 Lyon), France; 8 LUNAM University, Oniris, AMaROC Unit, Nantes, F-44307, France; 9 Department of Clinical Sciences, Swedish University of Agricultural Sciences (SLU), Uppsala, Sweden; 10 Department of Clinical Sciences of Companion Animals, Utrecht University, Utrecht, The Netherlands; University of Sydney, Australia

## Abstract

Autoinflammatory disease (AID) manifests from the dysregulation of the innate immune system and is characterised by systemic and persistent inflammation. Clinical heterogeneity leads to patients presenting with one or a spectrum of phenotypic signs, leading to difficult diagnoses in the absence of a clear genetic cause. We used separate genome-wide SNP analyses to investigate five signs of AID (recurrent fever, arthritis, breed specific secondary dermatitis, otitis and systemic reactive amyloidosis) in a canine comparative model, the pure bred Chinese Shar-Pei. Analysis of 255 DNA samples revealed a shared locus on chromosome 13 spanning two peaks of association. A three-marker haplotype based on the most significant SNP (*p*<2.6×10^−8^) from each analysis showed that one haplotypic pair (H13-11) was present in the majority of AID individuals, implicating this as a shared risk factor for all phenotypes. We also noted that a genetic signature (*F*_ST_) distinguishing the phenotypic extremes of the breed specific Chinese Shar-Pei thick and wrinkled skin, flanked the chromosome 13 AID locus; suggesting that breed development and differentiation has played a parallel role in the genetics of breed fitness. Intriguingly, a potential modifier locus for amyloidosis was revealed on chromosome 14, and an investigation of candidate genes from both this and the chromosome 13 regions revealed significant (*p*<0.05) renal differential expression in four genes previously implicated in kidney or immune health (*AOAH*, *ELMO1, HAS2* and *IL6*). These results illustrate that phenotypic heterogeneity need not be a reflection of genetic heterogeneity, and that genetic modifiers of disease could be masked if syndromes were not first considered as individual clinical signs and then as a sum of their component parts.

## Introduction

Autoinflammatory disorders (AIDs) result from the dysregulation of mediators of the innate immune system. These maladies cover a broad spectrum of diseases, from hereditary periodic fever syndromes defined by recurrent episodes of fever and inflammation with no known pathogenic or autoimmune cause, through to generalised multifactorial pathologies with an up-regulated innate immune response, such as gout and Crohn’s disease [1], [2]. The hallmark of many AIDs is the dysregulated secretion of the inflammatory cytokine interleukin (IL)-1β, and accordingly patient treatment and disease management has shifted to a broad application of IL-1 blockers [3], [4]. However it is not only the attacks of sterile fever and inflammation that are detrimental to patients. Persistent inflammation can lead to reactive amyloidosis, the accumulation of aberrantly produced acute phase proteins in multiple organs. As seen with hereditary fever patients, these aggregates are particularly damaging to the kidney and can result in organ damage, the need for transplantation or in the worst case scenario, organ failure and premature death [5].

In the recent past, multiple mouse models have been developed in order to study specific targets of AID pathway regulation; including knock-ins targeting inflammasome formation, the multiprotein oligomer required to cleave immature ILs prior to their cellular release, and knock-outs directed at specific ILs and their sensors (See [6] for a summary). Whilst these models have greatly enhanced the understanding of disease, they nonetheless represent the results of directed mutagenesis. We recently investigated the genetics of a spontaneous canine periodic fever model, Familial Shar-Pei Fever (FSF) in the pure bred Chinese Shar-Pei, and reported the involvement of a new gene in AID, Hyaluronan Synthase 2 (*HAS2*) [7]. This dog breed is strongly predisposed to many types of persistent inflammation in addition to recurrent fevers, including arthritis, Shar-Pei specific secondary dermatitis (hyaluronan filled vesicles affecting the skin, termed vesicular hyaluronosis, Figure S1), otitis and systemic reactive amyloidosis.

Shar-Pei can be recognized by their unique appearance with a heavily thickened and wrinkled skin, a trait that has been the target for strong artificial selection. The skin phenotype is called hereditary cutaneous hyaluronanosis (HCH) and resembles a condition also reported in humans [8], [9]. The degree of “wrinkledness” varies significantly between individuals, however the breed can be best described by three classes: a smooth, almost non-wrinkled traditional type; a less wrinkled bonemouth type and a heavily wrinkled meatmouth type which also has a padded muzzle. The Shar-Pei skin phenotype results from the excessive deposition of hyaluronan (HA) in the upper dermis of the skin [10] and in comparison with other dog breeds, Shar-Pei have an elevated HA production which is most likely due to an increased transcription rate of the HA synthesizing enzyme *HAS2* (measured in cultured dermal fibroblasts [8], [11]). In fact, by measuring serum HA as a proxy for HA production, it was possible to see 5–20 times more of this non-sulfated glycosaminoglycan (GAG) in Shar-Pei compared to other dog breeds [12].

HA represents one of the largest molecules in the body, not only by molecular weight (MW), but also in terms of the space it occupies, forming the main component of the extracellular matrix [13]. The diverse functions of HA are determined by a combination of MW and cellular concentration, with molecular homeostasis being maintained by high rates of synthesis and degradation [14], [15]. One intriguing feature of HA is its dual role in the inflammatory response. Low MW HA can serve as a danger associated molecular pattern (DAMP) and activate the inflammasome and the release of pro-inflammatory interleukins (pro-ILs) via two routes [16]. In one route, HA acts on toll-like receptors (TLR2 and 4), activating NF-κß to produce immature pro-ILs. The second pathway sees HA binding to the cellular receptor CD44, followed by two rounds of hyaluronidase cleavage, first at the membrane by HYAL2 and then in the lysosome by HYAL1, to produce the small oligosaccharides of HA which actually trigger inflammasome complex formation. Both routes must be activated in order for active cytokines to be released from the cell. As a counterpoint, high MW HA can actually prevent inflammasome formation through the blocking of cellular receptors and the alteration of cellular ion concentrations [17].

In the current investigation we were interested in delving further into Shar-Pei AID, this time with DNA from more than 250 individuals applied to a ten fold denser SNP panel. By breaking the syndrome into its constituent clinical signs, including fever, arthritis, otitis, vesicular hyaluronosis, amyloidosis, and by conducting discrete association analyses, we looked to answer if the same genetic cause was underlying each phenotype or, given the multi-factorial nature of disease, if modifying loci could be identified. With the clear link between the breed specific skin phenotype and FSF previously established [7], we also included a re-examination of the genetics of breed subtype in the current analysis.

## Results

### A Clear Genetic Signature of Phenotypic Selection within Shar-Pei

**Figure 1:**
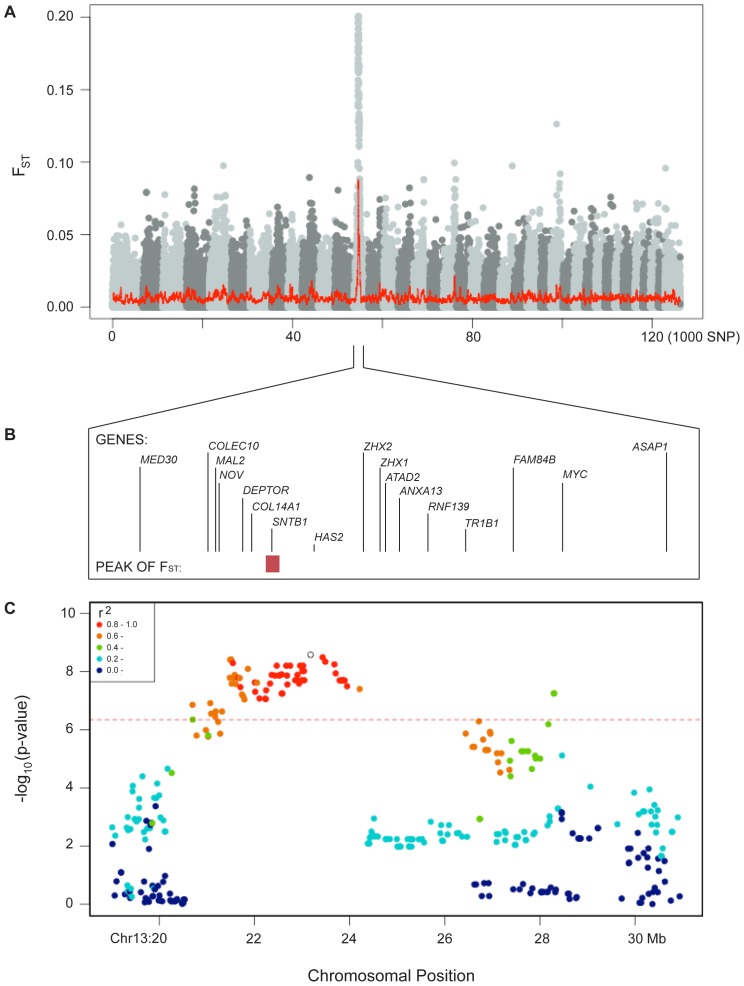
The genetic signature of within breed sub-type on chromosome 13. Individuals representing the extremes of physical appearance (Meatmouth, heavily wrinkled thickened skin, *n* = 123; Bonemouth, few wrinkles left into adulthood, *n* = 33) were compared. **A**. Pairwise *F*_ST_ was calculated between the two groups for 126,206 SNP and plotted in chromosomal order. Smoothed values in bins of 100 SNP are superimposed to better illustrate the peak of association (red line). **B**. A zoomed view of a subset of the genes from the chromosome region in relation to the peak of *F*_ST_ (based on most divergent SNP and a 5% threshold, red box). **C**. The plot of a mixed model association test for breed subtype. The top genome-wide significant SNP (13∶23180227, *p_MM_* = 2.63×10^−9^) has been highlighted with an open circle and linkage disequilibrium (*r^2^*) between this marker and the others in the region are coloured according to strength. A genome wide significant threshold (red dotted line) has been defined using an analysis specific Bonferroni 5% limit.

The individuals sampled in the current analysis span the phenotypic spectrum for Shar-Pei wrinkled and thickened skin. Whilst there is no quantitative measure for this trait, we used individuals from the two extremes as defined in an owner reported questionnaire. A comparison of 126,206 markers revealed a single smoothed *F*_ST_ peak on chromosome 13 (Figure 1A). Using the top ranked SNPs (*n* = 5, *F*_ST_ = 20.0%) to define this peak and then extending the range until the next consecutive SNP fell to the 5% level showed four distinct blocks (*CanFam* 2.0 chromosome 13 - Block 1∶22,467,697–22,586,357; Block 2∶22,666,702–22,748,362; Block 3∶22,854,585–23,004,428 and Block 4∶23,023,126–23,047,599). Between these, *F*_ST_ fell to below the genome average (*F*_ST_ = 0.64% SD 0.94%). These *F*_ST_ blocks encompass two genic features; canine *SNTB1* and a novel ribosomal RNA with unknown function, as well as the non-coding space downstream of *HAS2* (coded on the negative strand), a gene key to the production of the hyaluronan (HA) rich skin of Shar-Pei [8], [11]. The product of *SNTB1*, β1-syntrophin, has been implicated in both muscular dystrophies [18] and cholesterol homeostatis [19].

We also performed a mixed model (MM) Genome Wide Association Study (GWAS) (λ_MM SUBTYPE_ = 1.09) in order to better describe the breed subtype relationship. Only SNPs on chromosome 13 reached genome wide significance (Bonferroni *p*_MM_<4.53×10^−7^) and a plot across the region coloured for linkage disequilibrium (LD) shows two separate peaks separated by LD<0.8, but both with strong association with the trait (Figure 1C). The SNP most associated to breed subtype (BICF2G630613174∶23,180,227 bp; *p_MM_* = 2.63×10^−9^; *p_PERM_* = 9.99×10^−4^; open circle Figure 1C) was noted to be in high LD with all *F*_ST_ block SNPs (LD>0.8). The independent peak at 28.3Mb was supported by two genome wide significant SNP (BICF2P564681∶28,291,984 bp and BICF2S23051140∶28,301,987 bp; both *p_MM_* = 1.96×10^−7^; *p_PERM_* = 5.00×10^−3^). The expanded list of genes within this region which may be involved in breed subtype differentiation is reported in Table S1.

### Overlapping Genomic Association Patterns for all Inflammatory Phenotypes

**Figure 2:**
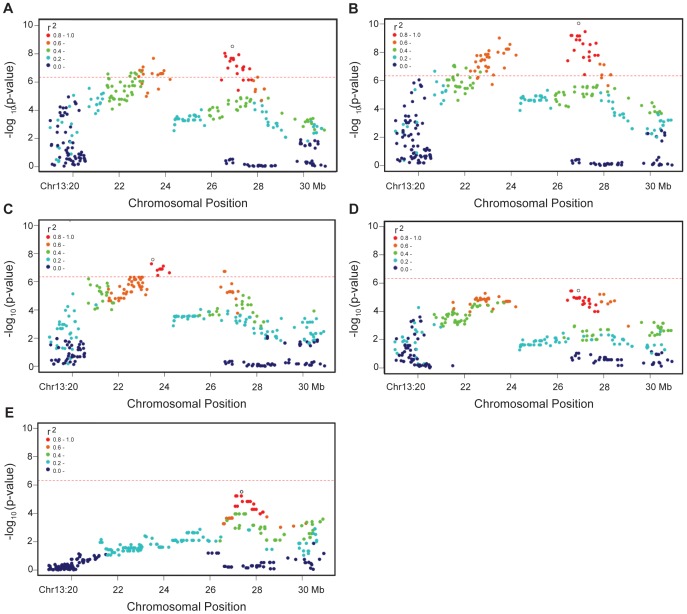
The five investigated signs of Shar-Pei autoinflammatory disease share overlapping association signals. The phenotypes of fever (**A**. *n* = 129), arthritis (**B**. *n* = 107), vesicular hyaluronosis (**C**. *n* = 46), and otitis (**D**. *n* = 27) were compared to a subset of healthy controls at least 7 years old (*n* = 24). The phenotype of amyloidosis (**E**) required individuals to undergo renal biopsy screening to be declared positive (*n* = 37) or negative (*n* = 14) for the deposits. In each panel the top genome-wide significant SNP has been highlighted with an open circle and linkage disequilibrium (*r^2^*) between this marker and the others in the region coloured according to strength. A genome wide significant threshold (red dotted line) has been defined using an analysis specific Bonferroni 5% limit.

The Shar-Pei breed is susceptible to a range of inflammatory conditions. In order to test if these are the result of a shared genomic locus, we examined each of five phenotypes (Fever, Arthritis, Vesicular Hyaluronosis, Otitis and Amyloidosis) in separate case/control GWAS. The number of individuals, SNPs passing quality control, genomic inflation factor before (λ_QT_) and after the application of a polygenic mixed model (λ_MM_), as well as the most significant SNP and the number of genome wide significant SNP are summarised for each analysis in Table 1. In each analysis, the most significant SNP (unfilled circle, Figure 2) was centered in one of two regions (21.5–24.2 Mb or 26.5–28.3 Mb) identified on chromosome 13. The analyses for Fever (Figure 2A) and Arthritis (Figure 2B) both reached genome wide significance and resulted in very similar patterns of association. This was perhaps not surprising given that a high fraction of Fever patients also suffer from Arthritis (93/129), however many more SNPs reached significance in the latter analysis (SNP_FEVER_ *n* = 37, SNP_ARTHRITIS_ *n* = 64). Comparing the pattern of association observed in these disease analyses to that revealed in the breed subtype GWAS (Figure 1B) showed clear overlap between the 23.5 Mb peak for all three phenotypes, however the LD block of SNPs at 21 Mb from breed subtype (orange) was not as tightly linked in disease. In addition, the most significant SNPs from the breed subtype analysis at 28.3 Mb are the final markers reaching genome wide significance in the Arthritis GWAS and were non-significant flanking markers in the Fever GWAS. We investigated the proportion of Meatmouth to Traditional individuals in each disease phenotype set to see if these results were reflective of breed membership in each analysis. While this ratio was slightly higher in the Arthritis analysis (Ratio_FEVER_ 5.06∶1; Ratio_ARTHRITIS_ 4.54∶1) and so may be in part responsible for the increased association observed at the ∼23 Mb region, the true peak of association for both Arthritis and Fever lies in the 28.3 Mb peak; indicating the genetics underlying breed subtype are not the key explanation of disease. Vesicular Hyaluronosis (Figure 2C) was the only other disease category to reach genome wide significance, and with a similar pattern to that observed previously.

**Table 1:** Summary of the separate GWAS analyses conducted on breed-subtype and five symptoms of Shar-Pei autoinflammatory disease (SPAID).

Phenotype Set	*n*	Control Set	*n*	Markers^1^	QT λ^2^	MM λ^3^	Top SNP	Position^4^	Sig^5^	SNP^6^
Breed Subtype Meatmouth	123	Breed Subtype Traditional	33	110,459	1.40 (9.6×10^−4^)	1.09 (8.1×10^−4^)	BICF2G630613174	23180227	2.63×10^−9^	67
Fever	129	Group 1	24	109,382	1.34 (9.1×10^−4^)	1.11 (6.4×10^−4^)	BICF2G630616424	26911739	2.97×10^−9^	37
Arthritis	107	Group 1	24	109,708	1.27 (1.1×10^−3^)	1.12 (9.1×10^−4^)	BICF2G630616424	26911739	8.99×10^−11^	64
VesicularHyaluronosis	46	Group 1	24	111,880	1.18 (4.8×10^−4^)	1.06 (4.4×10^−4^)	BICF2G630613491	23487992	2.69×10^−8^	13
Otitis	27	Group 1	24	108,396	1.24 (2.6×10^−4^)	1.03 (2.3×10^−4^)	BICF2G630616424	26911739	3.38×10^−6^	0
Amyloidosis Positive	37	AmyloidosisNegative	14	106,298	1.36 (1.1×10^−4^)	1.04 (9.5×10^−5^)	BICF2G630616702	27371905	3.00×10^−6^	0

1 Number of markers from a genotyped set of 173,662 which passed two rounds of quality control.

2 The genomic inflation factor measured from the unadjusted qtscore GWAS.

3 The genomic inflation factor after the application of a polygenic mixed model encompassing the Identity By State (IBS) matrix.

4 Genomic position (*CanFam* 2.0, chromosome 13, bp) of the top SNP as ranked by ^5^Significance of mixed model p-value.

6 Number of SNP which exceeded the Bonferroni 5% threshold for significance.

The LD pattern of Otitis (Figure 2D) matched those of the previous phenotypes, but interestingly, the Amyloidosis (Figure 2E) analysis showed that the link between the left and right peaks had been severed. Within Amyloidosis cases, 90% also suffered from Fever, 65% from Arthritis but only 18% had been diagnosed with all three conditions. Amyloidosis is the only AID phenotype set in the analysis which has a dedicated control set. Individuals were required to have both a clinical history free from unexplained inflammation, and to have also tested negative for amyloid deposits in renal biopsies. This binary diagnosis of disease leaves no margin for interpretation and may explain why the Amyloidosis peak almost reached genome wide significance, whilst the Otitis peak with more individuals, did not.

In order to reveal additional masked associations, we also investigated a second polygenic model for each GWAS which included the most significant SNP in a conditional analysis. In all cases this lead to a deflation in lambda (λ = 0.97–0.94) and no new significant peaks (data not shown). For the phenotype sets of Fever, Arthritis, Vesicular Hyaluronosis and Otitis we also conducted GWAS with Health Control Set 2. By relaxing the age limitation (Control Group 1: *n = *24, average age = 9.75±1.96; Control Group 2: *n = *36, average age = 8.36±2.58) we were able to recruit more individuals into the study. The result was generally the same i.e. GWAS which had reached genome wide significance previously did so again, however whilst the peak of association did not shift, the number of SNP which were seen to be significant fell (Table S2).

We decided to use the top SNPs (C<BICF2G630613491<T, A<BICF2G630616424<G, A<BICF2G630616702<G) from these significant GWAS results to build a haplotype spanning this segment of chromosome 13. This allowed us to assess the degree of allele sharing at this locus across all phenotype sets. Of the 16 pairs of phased haplotypes predicted from the total data set, haplotype pair H13-11 (CGA/CGA) was predominant in all disease classes with between 70.5–85.2% of affected individuals carrying this locus (Table 2). This locus was also moderately observed in healthy individuals (Health Group 1, 8.3%; Amyloidosis Negative, 14.3%). The main haplotype pairs in healthy individuals were H13-17 (CGA/TAG: Health Group 1, 33.3% and Amyloidosis Negative, 42.9%) and H13-37 (CAG/TAG: Health Group 1, 16.7% and Amyloidosis Negative, 28.6%). The latter was rare in the diseased data set, appearing in only the Vesicular Hyaluronosis data set (H13-37 = 2.2%). Taken together, this suggests a shared haplotypic pair (H13-11) for all disease sets, and two potentially protective sets (H13-17 and H13-37). A list of all genome-wide significant SNPs, their closest gene and their membership to each phenotypic symptom class is given in Table S2.

**Table 2:** Major haplotype pairs observed across chromosome 13 as defined by GWAS significant SNP.

	Breed Type		Disease Sets				Health Sets		
Haplotype^1^	Traditional	Meatmouth	Fever	Arthritis	Vesicular Hyaluronosis	Otitis	Amyloidosis Positive	Amyloidosis Negative	Control Set 1
H13-11[CGA/CGA]	0.303	0.724	0.705	0.757	0.761	0.852	0.703	0.143	0.083
H13-13[CGA/CAG]	0.000	0.049	0.047	0.028	0.022	0.037	0.108	0.000	0.000
H13-17[CGA/TAG]	0.152	0.114	0.147	0.131	0.109	0.074	0.081	0.429	0.333
H13-37[CAG/TAG]	0.091	0.008	0.000	0.000	0.022	0.000	0.000	0.286	0.167
H13-77[TAG/TAG]	0.121	0.000	0.008	0.000	0.000	0.000	0.000	0.000	0.042
Other HaplotypePairs^2^	0.333	0.106	0.093	0.084	0.087	0.037	0.108	0.143	0.375

1 Phased haplotypes are composed of the top SNP identified in each GWAS where genome-wide significance was reached and estimated from the total population frequency.

2 Haplotype pairs observed at frequencies less than 10% in a sample set have been collapsed.

**Figure 3:**
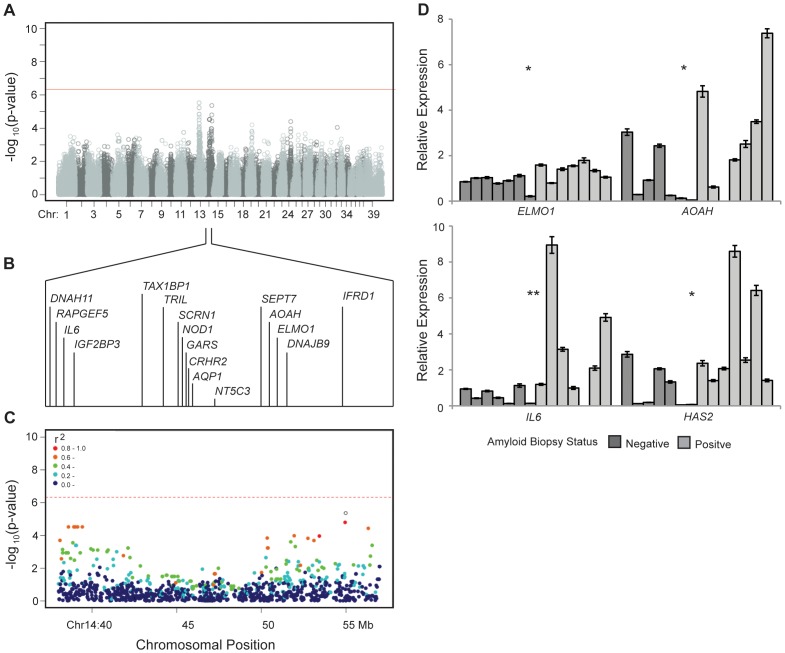
A novel locus on chromosome 14 is associated with Shar-Pei amyloidosis. A . The genome wide association study of amyloidosis positive (*n* = 37) and negative (*n* = 14) individuals revealed a signal of association on chromosome 14 (14∶54948811, *p_MM_* = 4.34×10^−6^) which was of slightly reduced strength to that observed on chromosome 13 (13∶27371905, *p_MM_* = 3.0×10^−6^). **B**. A zoomed view of a subset of the genes across the chromosome 14 disease associated region, **C**. This region encompasses two peaks in moderate LD (*r^2^* = 0.6) as indicated by heat colouring. A genome wide significant threshold (red dotted line) has been defined using as Bonferroni 5%. **D**. The scaled plots of genes demonstrating differential expression between amyloidosis negative (*n* = 7) and positive (*n* = 7) kidney biopsies. Samples are shown in the same order for each gene and also in order of phased H14 haplotypes (Table S3). Each sample registered expression, although in some cases it was negligible. p-values 0.05>0.01, *>**.

Whilst not reaching genome wide significance, the third most significant SNP in the GWAS for Amyloidosis was actually on chromosome 14 (Figure 3, *p_MM_* = 4.34×10^−6^), suggesting that in addition to the chromosome 13 signal, there may be additional modifying loci. Investigation of the top SNPs from chromosome 14 revealed two main regions of association at approximately 39 Mb and 55 Mb. The eight most significant SNPs [BICF2P1345913, TIGRP2P191729, BICF2P1033504, BICF2P354070, BICF2P1275408, BICF2P321096, BICF2P765917, BICF2S23510954; *p_MM_* = 3.72×10^−5^–4.34×10^−6^] were used to define a haplotype spanning the region (Table 3). Phasing the data revealed 26 haplotype pairs in the population, although most were rare. Amyloidosis cases predominantly carried pair H14-11 (TGGGGCCC/TGGGGCCC: *n = *32/37, 86.5%) however this was ranked equal second in Amyloidosis controls (*n = *2/14, 14.3%) behind H14-111 (TGGGGCCC/CATCATAT: *n = *3/14, 21.4%).

**Table 3:** Distribution of chromosome 14 Amyloidosis haplotype pairs in the total population of Shar-Pei analysed.

Haplotype^1^	Amyloidosis Positive	Amyloidosis Negative
H14-11 [TGGGGCCC/TGGGGCCC]	0.865	0.143
H14-15 [TGGGGCCC/TGGGGTAT]	0.027	0.143
H14-111 [TGGGGCCC/CATCATAT]	0.000	0.214
H14-1111 [CATCATAT/CATCATAT]	0.000	0.143
Other Haplotype Pairs^2^	0.108	0.357

1 Phased haplotypes are composed of the top eight SNP identified from the chromosome 14 and are estimated from the total population frequency (*n = *255).

2 Pairs observed at frequencies less than 10% in a sample set have been collapsed.

### An Amyloidosis Candidate Gene Approach Reveals Renal Differential Expression

Access to renal tissue from individuals screened for amyloidosis (positive, *n* = 7; negative, *n* = 7), but not available to the GWAS, prompted a candidate gene expression study guided by the association results. Multiple genes located in the two chromosome 14 peaks, and also from the genomic region between these, have previously been implicated in human nephropathic and immune disease states. These included Interleukin 6, a potent activator of inflammation and the secretion of amyloid protein A (*IL6*; 14∶39,432,809–39,437,123) and Interferon-related developmental regulator 1, involved in inflammatory signaling (*IFRD1*; 14∶54,994,707–55,037,998). Of the 21 genes tested, four showed differential expression, with Acyloxyacyl Hydrolase (*AOAH*), Engulfment and Cell Motility 1 (*ELMO1*), Hyaluronan synthase 2 (*HAS2*) and Interleukin 6 (*IL6*) all showing higher expression in the renal samples from amyloidosis positive individuals (Figure 3D). Each of these genes has either a clear immunological role or a role in organ viability; *AOAH* prevents extended host inflammatory responses to endotoxins [20], *ELMO1* is essential to the phagocytosis of apoptotic cells [21] and *HAS2* acts as a rheostat during inflammation [22].

Where possible, the haplotypic phase of each biopsied individual was also assessed. Most were predicted to be H14-11 (*n* = 5/12; Table S3), which was not unexpected given that this was the dominant population wide haplotype (*n* = 137/255). An analysis of gene expression relative to haplotype did not reveal any significant results, but was restricted to a small sample size (Table S3).

## Discussion

A concerted effort was made in the current study to collect individuals that spanned both the phenotypic breed spectrum as well as those for which rigorous clinical classifications could be made. We paid particular attention to the signs of persistent recurrent inflammation, expanding our understanding of classical Familial Shar-Pei Fever (FSF) to more thoroughly encompass the clinical signs of unexplained fever, arthritis, vesicular hyaluronosis, otitis and amyloidosis. This expanded clinical spectrum allowed us to more accurately apply the term of *Shar-Pei Autoinflammatory Disease* (SPAID) to define this syndrome.

The pathological outcomes of autoinflammatory disease (AID) derive from a dysregulation of the innate immune response. In our first examination of Shar-Pei and FSF using 44 individuals and 17,000 SNPs, we revealed a 12 Mb signal of interspersed breed homozygosity and disease association on chromosome 13 (22–34 Mb) which encompassed Hyaluronan Synthase 2 (*HAS2*) [7]. We proposed a role for *HAS2* over-expression in driving the amplified production of hyaluronan (HA) and in turn, driving this canine AID state. The increased HA observed in the dermis of this breed is inherently linked to the development of the skin phenotype, but fragmented HA can also be seen as a *self* trigger of the innate immune response, leading to the release of proinflammatory cytokines through danger associated molecular pattern (DAMP) recognition and inflammasome activation.

The present study, with 255 individuals and an order of magnitude more markers, allowed this interval to be further refined, revealing two clear signals on chromosome 13, one at ∼22–24 Mb and the other between ∼27–29 Mb. Each clinical phenotype and the breed subtypes were treated as independent variables and assessed with a combination of genetic fixation (Breed subtype *F*_ST_) and genetic association. The clear co-localisation of the genetic signal for breed subtype with the peaks of association for each of the SPAID phenotypes shows the dual outcomes artificial selection has played in forming the modern Shar-Pei breed.

The chromosome 13 signal for Shar-Pei “wrinkled skin” has been observed by others at slightly different resolutions due to varying marker densities and sample cohorts [23]. However, this pattern of within breed genetic fixation is not unique and can be seen as a hallmark of domestic animal breeding; attributed to assortative mating practices and the use of popular dams and sires to rapidly produce and improve genetic gains [24], [25]. We note that the *F*_ST_ signal marking breed subtype differentiation (22.4–23 Mb) actually spans the gene β1-syntrophin (*SNTB1*). While *SNTB1* is predominantly recognised for its interaction with dystrophin [18], it has also been shown to have a critical role in cholesterol homeostasis through its interaction with ATP-binding cassette transporter A1 (*ABCA1*) [18]. In human primary skin fibroblasts, the silencing of *SNTB1* lead to a 50% decrease in cellular cholesterol efflux, whilst increased gene expression resulted in the altered cellular distribution and activity of the ABCA1 protein [19]. It could be intriguing to hypothesize that breed differentiation is not simply a matter of HA over-production (Shar-Pei compared to other breeds [8], [11]–[12]), but that there are further as yet unseen physiological changes which mark the distinction between meatmouth and bonemouth individuals. Within the larger genomic region implicated in breed subtype differentiation through the GWAS, there are two genes involved in collagen production and recognition (*COL14A1*, *COLEC10*) as well as a raft of genes essential for immunological response (including T-cell differentiation protein 2, *MAL2*; DEP domain containing MTOR-interacting protein, *DEPTOR*), kidney function (family with sequence similarity 84, member B, *FAM84B*) and constitutive gene expression (v-myc avian myelocytomatosis viral oncogene homolog, *MYC*). These later genes are of particular interest given the overlap between breed subtype genetic selection and the high level of SPAID predisposition in the Shar-Pei (as FSF in [26]).

By dividing SPAID into its component clinical signs, we were able to see that the highest peak of association for both fever and arthritis (27.5–29 Mb) was in strong, but not perfect LD with the peak containing *HAS2* (24 Mb, *r^2^* = 0.6–0.8, Figure 2A and 2B). However, given the significant genomic association of the 24 Mb region, it is likely that variants residing in this peak are still involved in disease. Inspection of the genome-wide significant SNPs from each phenotype GWAS showed a clear overlap in associated alleles (Table S2), and this was formalised through designation of a disease haplotype (H13-11, Table 2), observed in more than 70% of individuals with SPAID.

A closer examination of the Amyloidosis GWAS results showed one distinct peak of association on chromosome 13 that was centered on *FAM84B* and *MYC*, both of which are known to be expressed in renal tissue. Shar-Pei amyloidosis is reactive in nature and results in the deposition of amyloid AA protein most commonly in the kidneys; but it can also be systemic, with a wide tissue distribution including the heart, spleen, liver, adrenal glands, pancreas and intestinal submucosa [27]. Shar-Pei amyloidosis is typically described as involving the renal medulla, which places it in stark contrast with the glomerular disease most commonly described in other canines and mammals (not including felines), however diffuse glomerular amyloidosis is not uncommon [27], [28].

We also noted a signal of association to amyloidosis on chromosome 14, suggestive of a multi-factorial mode of disease and the presence of genetic modifiers for this SPAID subphenotype. Closer inspection revealed two peaks 16 Mb apart and in low LD with each other (*r^2^* = 0.6–0.8, Figure 3B). An examination of the genes encased by these peaks revealed the enrichment again for those involved in both immunological and renal health, of particular note in each flank were Interleukin 6 (*IL-6*) and Interferon-related developmental regulator 1 (*IFRD1*). Elevated levels of IL-6 have been shown to be associated to FSF [26], and whilst this is likely a reflection of IL-1ß cytokine induction [29], IL-6 is itself a potent trigger of amyloid A protein [30], making it an obvious candidate gene for further study. Likewise, *IFRD1* is shown to act as a positive cofactor of *MyoD* and repress the transcriptional activity of *NF-κß* [31], this is of interest since low molecular weight HA can actually serve to promote *NF-κß* transcription.

Our targeted examination of renal gene expression was of modest size (*n* = 14) but did offer insight into the immune processes of this organ, with *HAS2*, *AOAH*, *ELMO1* and *IL6* all showing higher expression in amyloid positive tissue (Figure 3D). As we have noted previously [7], hyaluronan (HA) synthesized by *HAS2*, is able to activate the two pathways required for the release of mature pro-inflammatory interleukins from the cell, namely i) *NF-κß* transcription following binding to either TLR2 or 4 and ii) inflammasome formation through lysomome triggered activation. Continuous, cyclic production of HA could lead to chronic states of inflammation. This resultant cytokine cascade can lead to an acute phase response, and an up to 1000-fold increase of hepatic serum amyloid A (SAA) production [32]. Circulating SAA is associated with high-density lipoprotein_3_ in blood [33], and in pathogenic states deposits in the kidney as the main component of inflammatory driven amyloid A (AA) amyloidosis. This acute phase protein is itself a key modulator of the innate immune response and like HA, can provide the two signals required for the inflammasome mediated release of mature interleukins. SAA activates the *NF-κß* pathway in the same was as HA, through direct binding with TLR4, but its activation of the inflammasome is via a different route, binding with the cellular receptor P2X [34], [35].

In the above examples, HA and SAA are acting as Danger Associated Molecular Patterns (DAMPs) to trigger the formation of the inflammasome. This multiprotein complex is also formed in response to Pathogen Associated Molecular Patterns (PAMPs) such as lipopolysaccharides (LPS), and in that case the release of pro-inflammatory cytokines is modulated by the to the feedback production of a host lipase, acyloxyacyl hydrolase (AOAH) [20], [36]. AOAH, encoded by *AOAH*, deacylates LPS which not only reduces the available active PAMP substrate, but also serve to compete for available TLR4 receptor binding [37]. It is likely that the amyloid positive tissue is mounting a DAMP/PAMP regulation response through increased *AOAH* expression, but that remains to be formally tested.

We also noted a significant increase in the expression of *IL6* in amyolid positive tissue. This could be a direct downstream reaction to HA and SAA driven IL-1ß production [29], [38], although it should be noted that SAA can also play a chemotactic role to macrophages, neutrophils and mast cells, leading to further *IL6* expression. This draw of macrophages into kidney tissue directly links to *ELMO1* expression, as this gene is essential to macrophage engulfment of apoptotic cells [39]. We believe that a more comprehensive expression study in kidney, as well as other tissues, could provide more insight into the action of amyloidosis. Whilst some of the gene expression patterns we observed could be a reaction to disease rather than the direct or modifying cause, an individual’s genetic predisposition to DAMP/PAMP stress response may directly impact their susceptibly to amyloidosis. This hypothesis will be especially relevant in future as it is hoped more DNA samples will become available to (i) provide increased power to strengthen the GWAS, (ii) allow for the further development and evaluation of H14-risk and protective haplotypes and ultimately, (iii) to allow for the identification of the causal variants residing in this modifying locus.

SPAID is only one example of a disease which has been inadvertently enriched for in a population through the selective breeding for a desired trait. A classic example from dog genetics is the predisposition to dermoid sinus of Ridgeback dogs from the duplication of a 133 kb region encompassing four genes on chromosome 18 [40]. However, in that case it was a dominant mutation with a definitive phenotype. The genetic transmission of SPAID is more complex, underlined by the fact that individuals from the same family need not present with the same subset of symptoms to fall into the autoinflammatory syndrome catchment. The dissection of genetic risk factors from the chromosome 13 and 14 loci, and their interactions, is essential in order to understand the aetiology of this syndrome in Shar-Pei dogs. An increased understanding of the mechanisms underlying autoinflammatory disease predisposition and progression will also serve to offer insight into innate immune system of other mammalian species, including humans.

## Materials and Methods

### Ethics Statement

Purebred Shar-Pei pet dogs were sampled from France, the Netherlands, Sweden and the United States following owner consent and ethical approvals (DEC Utrecht University 10813, SLU Dnr. C103/10, MIT 0910-074-13).

### Animal Material

The clinical history of all dogs (*n* = 255) was assessed using owner completed health questionnaires, carefully reviewed medical records and when possible in conjunction with the veterinarian. Each individuals sex, geographic origin, health status and breed subtype has been summarised in Table S4. For the health Genome Wide Association Studies (GWAS) five clinical signs of inflammation (Fever, Arthritis, Vesicular Hyaluronosis, Otitis and Amyloidosis; see below) were used to define the five case- and three control-subsets. The number of individuals within each case and control subset varies (from *n* = 129 to *n* = 14) and membership to a case set is not necessarily exclusive. The latter can be visualised with the aid of a Venn diagram (Figure S2).

1. Fever (*n* = 129): Recurrent bouts of fever lasting 6–72 hours with no underlying infection.
2. Arthritis (*n* = 107): Recurrent or prolonged bouts of joint (hock) inflammation with no known underlying infection.
3. Vesicular Hyaluronosis (*n* = 46): Dermatological vesicular changes to the skin leading to recurrent or persistent secondary inflammation (Figure S1).
4. Otitis (*n* = 27): Recurrent or chronic inflammation of the ears.
5. Amyloidosis (*n* = 37): Congo Red stained amyloid deposits observed in a *post mortem* kidney biopsy.
6. Control Group 1 (*n* = 24): Strictest control class. Individuals seven years or older, with no clinical history of unexplained inflammation.
7. Control Group 2 (*n* = 36): Relaxed control class. Individuals older than four years with no clinical history of unexplained inflammation.
8. Control Group 3 (*n* = 14): No Congo Red stained amyloid deposits observed in a *post mortem* kidney biopsy and no clinical history of unexplained inflammation.

Two further groups were defined based on the physical appearance of the individual as described by the owner. These were classed as Meatmouth (*n* = 123), heavily wrinkled, thicken skin and Bonemouth (*n* = 33), at the opposite end of the physical spectrum.

### Genotyping, Data Quality Control and Genetic Analysis

DNA was genotyped using the Illumina Canine 170 K SNP-Chip. Breed subtype pair-wise SNP *F*_ST_ analyses were performed using PLINK v1.07 [41] for SNP pruning (call rate >99% and minor allele frequency >0), and calculation of allele frequencies, whilst SNP-specific and smoothing *F*_ST_ calculations (moving average of 100 SNP) were performed in R v2.15.0 following the methodology of [42]. The extent of the selected region was defined as the top marker plus consecutive markers until those ranking outside of the top 5% were encountered. GWAS analyses were performed using GenABEL v1.7-2 and cgmisc packages in R v2.15.0. For each case/control analysis, two rounds of quality control were employed. The first included tests for missing genotype calls for single SNP and individuals (<5%), minor allele frequency (<0.05) and strong deviations from Hardy-Weinberg Equilibrium (HWE, p>1×10^−8^). The second controlled for HWE deviations in controls only using a FDR level of 0.2. From the initial 173,662 SNP genotyped, this left between 106,298 and 111,880 markers for further analysis (Table 1). The genetic relationship between cases and controls was reported using (i) multiple dimension scaling plot of the *Identity-By-State* (IBS) matrix based on 2000 autosomal markers, weighted for allelic frequency, (ii) quantile-quantile plot for GWAS observed versus expected p-values, (iii) estimate of genome-wide data inflation (λ). Basic Cochran-Armitage trend tests for association (qtscore) revealed evidence of stratification (λ>1.0) in each of the GWAS considered, so for all analyses a polygenic mixed model was fitted which encompassed the IBS matrix. SNPs exceeding 5% Bonferroni genome wide significance were also permutated to account for multiple testing (100,000 iterations). Measures of linkage disequilibrium (*r^2^*) were estimated using the r2fast function in GenABEL v1.7-2. Haplotypic phase was estimated using the full population data set (*n* = 255) and PHASE v2.1.1 [43], [44] with 1000 iterations, a thinning interval of 10 through the Markov chain and a burn-in of 1000. The process was repeated five times, reporting the identical most likely pairs in each run.

### Gene Expression Analysis

Fourteen individuals with a Congo Red renal diagnosis of amyloidosis [45] also had kidney tissue stored in RNAlater (Qiagen). Total RNA was extracted from approximately 50 milligrams of tissue using the RNeasy mini kit (Qiagen) following the manufacturers instruction and with an additional Turbo DNA-free treatment (Ambion) to remove genomic DNA (gDNA). RNA was quantified on a Bioanalyser (RIN between 7 and 9 for all samples) prior to cDNA synthesis using one microgram of total RNA and the Advantage RT-for-PCR kit (Clontech). No gDNA contamination was identified and so the expression of 23 genes was measured (19 candidate genes from *Cfa*14, two from *Cfa*13 and two housekeeping genes, Table S5) using SYBR Green (LifeTechnologies) chemistry and a HT7900 instrument (LifeTechnologies) Candidate genes were selected based on their position relative to the key associated SNP for the Amyloidosis GWAS and/or known roles in kidney or immune function. Expression values for each target and reference product were calculated using primer specific amplification efficiencies prior to normalisation using geNorm [46] as implemented in qbasePLUS (Biogazelle). Two-sided Mann-Whitney tests were used to assess the significance of differential expression between amyloidosis positive (*n* = 7) and negative sample (*n* = 7) groups.

## Supporting Information

Figure S1 **Examples of vesicular hyaluronosis.**
(DOCX)

Figure S2 **Overlapping membership of individuals to each of the five symptoms of Shar-Pei Autoinflammatory Disease (SPAID).**
(DOCX)

Table S1 **Summary of four SPAID symptoms GWAS analysed with a relaxed control group.**
(DOCX)

Table S2 **Summary of the significant SNP from chromosome 13 for the GWAS which reached genome-wide significance.**
(DOCX)

Table S3 **Individuals used for chromosome 14 candidate gene study.**
(DOCX)

Table S4 **Binary phenotypic description of each individual included in the study.**
(DOCX)

Table S5 **Primer list for candidate gene expression.**
(DOCX)
