# Supplementary material for: Thorough Investigation of a Canine Autoinflammatory Disease (AID) Confirms One Main Risk Locus and Suggests a Modifier Locus for Amyloidosis
Source: PLoS One. 2013 Oct 9;8(10):e75242. doi: 10.1371/journal.pone.0075242 (PMC3793984; doi:10.1371/journal.pone.0075242)
Supplement: Figure S1 — Examples of vesicular hyaluronosis. (DOCX) [file pone.0075242.s001.docx]

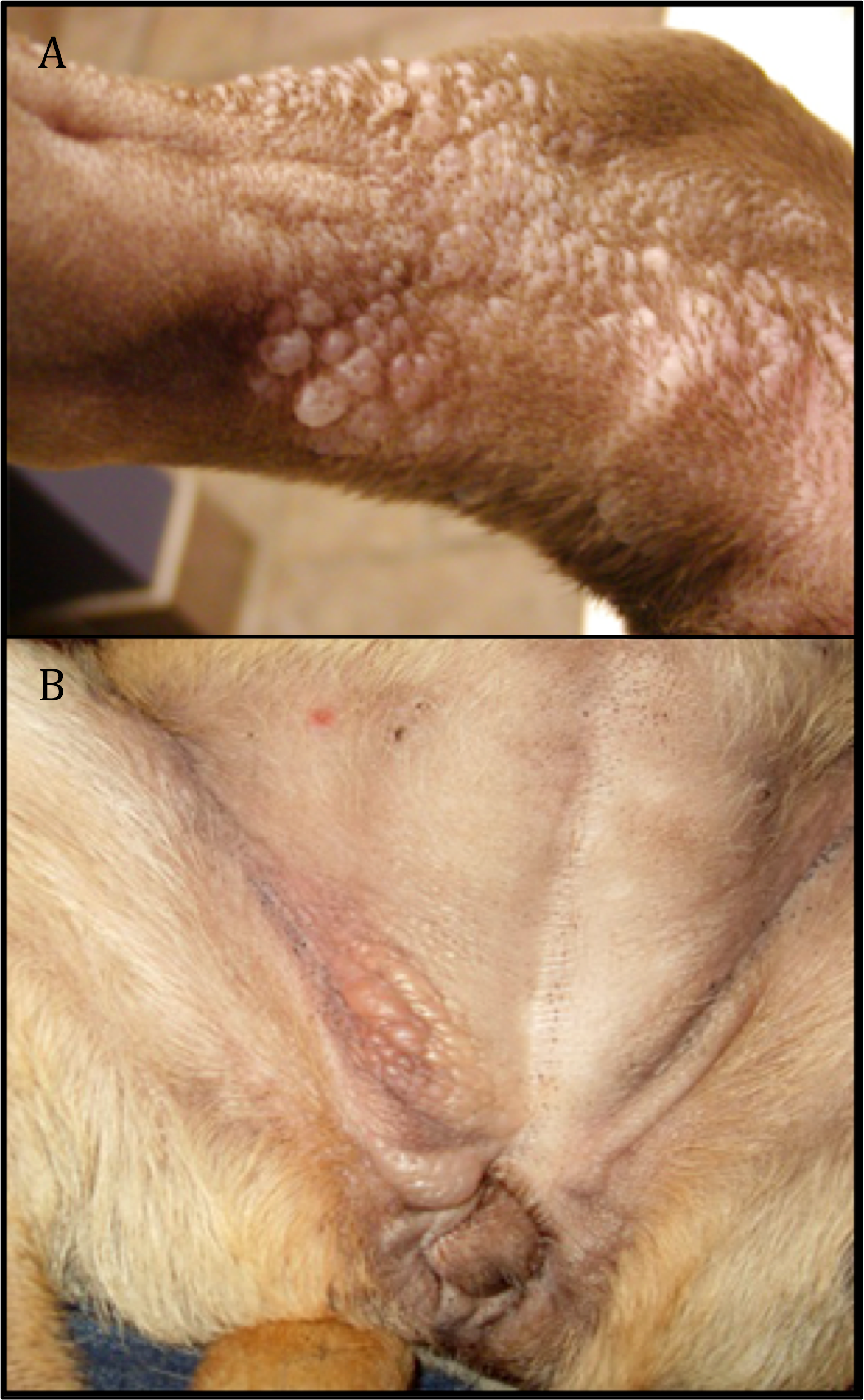


**Figure S1. Examples of vesicular hyaluronosis.** The Shar-Pei breed specific dermatological changes display as vesicles on the surface of the skin. Manifestations are typically seen on the (**A**) upper legs, (**B**) belly, chest, ventral neck and flanks. The vesicles are filled with hyaluronan and easily rupture which can lead to secondary infections.
