# Supplementary material for: Thorough Investigation of a Canine Autoinflammatory Disease (AID) Confirms One Main Risk Locus and Suggests a Modifier Locus for Amyloidosis
Source: PLoS One. 2013 Oct 9;8(10):e75242. doi: 10.1371/journal.pone.0075242 (PMC3793984; doi:10.1371/journal.pone.0075242)
Supplement: Figure S2 — Overlapping membership of individuals to each of the five symptoms of Shar-Pei Autoinflammatory Disease (SPAID). (DOCX) [file pone.0075242.s002.docx]

**
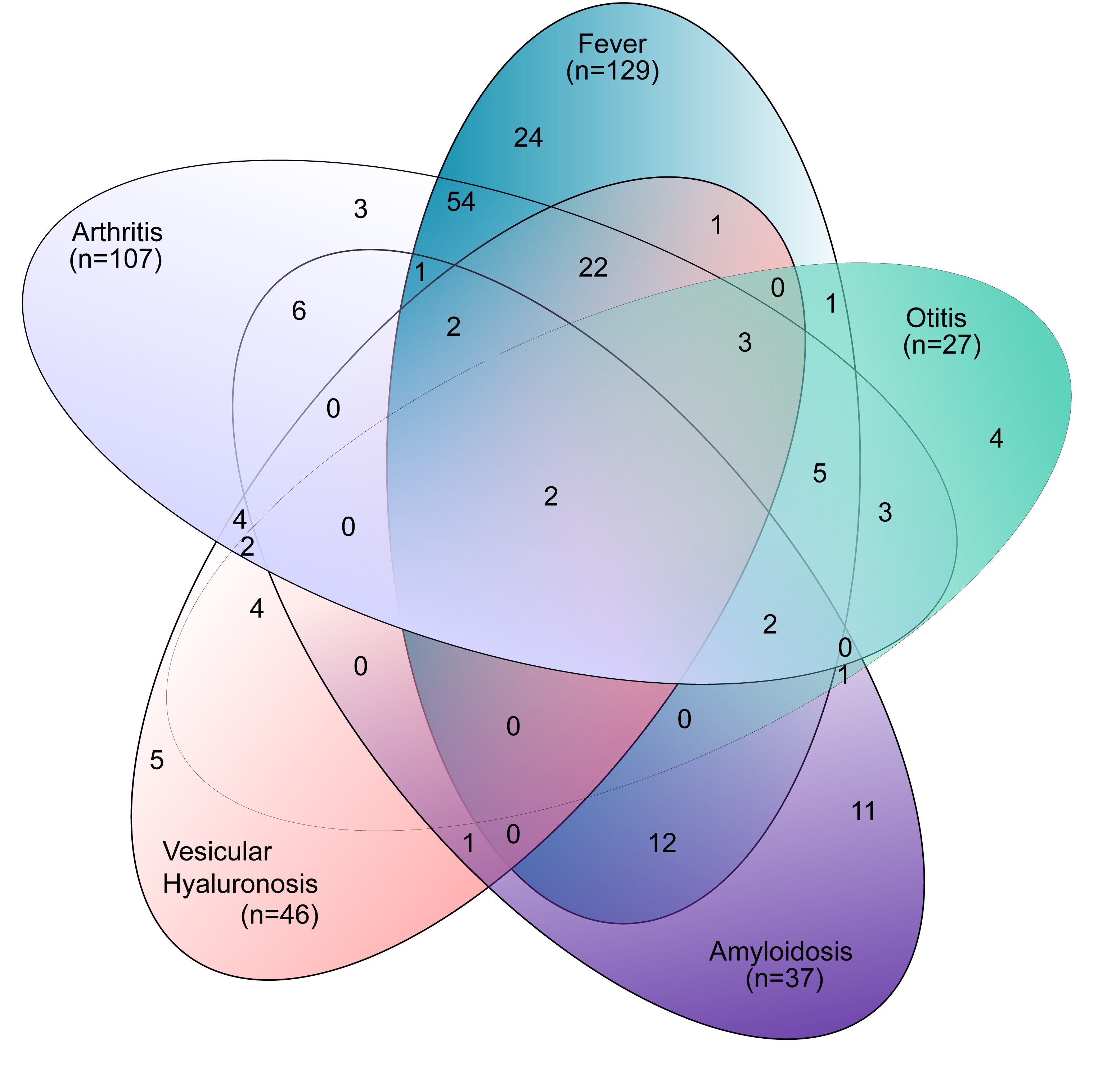
**

**Figure S2. Overlapping membership of individuals to each of the five symptoms of Shar-Pei Autoinflammatory Disease (SPAID).** The majority of individuals belong to two or more groups with only two individuals diagnosed to suffer from all symptoms.
