# Supplementary material for: Thorough Investigation of a Canine Autoinflammatory Disease (AID) Confirms One Main Risk Locus and Suggests a Modifier Locus for Amyloidosis
Source: PLoS One. 2013 Oct 9;8(10):e75242. doi: 10.1371/journal.pone.0075242 (PMC3793984; doi:10.1371/journal.pone.0075242)
Supplement: Table S1 — Summary of four SPAID symptoms GWAS analysed with a relaxed control group. (DOCX) [file pone.0075242.s003.docx]

**Table S1. Summary of four SPAID symptoms GWAS analysed with a relaxed control group.**

| Phenotype Set | *n* | Control Set | *n* | Markers^1^ | QT λ^2^ | MM λ^3^ | Top SNP | Position^4^ | Significance^5^ | SNP^6^ |
| --- | --- | --- | --- | --- | --- | --- | --- | --- | --- | --- |
| Fever | 129 | Group 2 | 36 | 109,392 | 1.39 (8.5x10^-4^) | 1.07 (4.4x10^-4^) | BICF2G630616424 | 13:26911739 | 1.02x10^-8^ | 17 |
| Arthritis | 107 | Group 2 | 36 | 109,622 | 1.31 (1.0x10^-3^) | 1.08 (7.1x10^-4^) | BICF2G630616424 | 13:26911739 | 2.49x10^-10^ | 37 |
| Vesicular Hyaluronosis | 46 | Group 2 | 36 | 109,399 | 1.18 (4.1x10^-4^) | 1.05 (3.8x10^-4^) | BICF2G630613491 | 13:23487992 | 1.10x10^-7^ | 11 |
| Otitis | 27 | Group 2 | 36 | 108,362 | 1.22 (2.2x10^-4^) | 0.99 (1.4x10^-4^) | BICF2G630617043 | 13:27831739 | 6.50x10^-6^ | 0 |

^1^Number of markers from a genotyped set of 173,662 which passed two rounds of quality control. ^2^The genomic inflation factor measured from the unadjusted qtscore GWAS. ^3^The genomic inflation factor after the application of a polygenic mixed model encompassing the Identity By State (IBS) matrix. ^4^Genomic position (CanFam 2.0 chromosome:base pair) of the top SNP as ranked by ^5^mixed model p-value. ^6^Number of SNPs which exceeded the Bonferroni 5% threshold for significance.
