# Supplementary material for: Thorough Investigation of a Canine Autoinflammatory Disease (AID) Confirms One Main Risk Locus and Suggests a Modifier Locus for Amyloidosis
Source: PLoS One. 2013 Oct 9;8(10):e75242. doi: 10.1371/journal.pone.0075242 (PMC3793984; doi:10.1371/journal.pone.0075242)
Supplement: Table S2 — Summary of the significant SNP from chromosome 13 for the GWAS which reached genome-wide significance. (DOCX) [file pone.0075242.s004.docx]

**Table S2. Summary of the significant SNP from chromosome 13 for the GWAS which reached genome-wide significance.**

| SNP | Position (bp) | Allele | Phenotype^1^ | Nearby Gene^2^ | Strand | Distance^3^ (bp) |
| --- | --- | --- | --- | --- | --- | --- |
| BICF2G630611131 | 20695492 | G/A | B | *SAMD12 (HS)* | - | 234364\|\|-142539 |
| BICF2G630611144 | 20700683 | C/T | BA | *SAMD12 (HS)* | - | 229173\|\|-147730 |
| BICF2P648415 | 21069059 | T/C | B | *SAMD12 (HS)* | - | -139203\|\|-516106 |
|  |  |  |  | *TNFRSF11B (HS)* | + | 147065\|\|119417 |
| BICF2G630611295 | 21086488 | G/C | B | *SAMD12 (HS)* | - | -156632\|\|-533535 |
|  |  |  |  | *TNFRSF11B (HS)* | + | 129636\|\|101988 |
| BICF2G630611413 | 21171455 | G/A | B | *SAMD12 (HS)* | - | -241599\|\|-618502 |
|  |  |  |  | *TNFRSF11B (HS)* | + | 44669\|\|17021 |
| BICF2G630611429 | 21183942 | A/G | B | *SAMD12 (HS)* | - | -254086\|\|-630989 |
|  |  |  |  | *TNFRSF11B (HS)* | + | 32182\|\|4534 |
| BICF2G630611546 | 21315435 | G/C | B | *TNFRSF11B (HS)* | + | -99311\|\|-126959 |
|  |  |  |  | *COLEC10 (HS)* | + | 8558\|\|47275 |
| BICF2G630611756 | 21480231 | A/C | B | *MAL2 (HS)* | + | -36103\|\|-25452 |
|  |  |  |  | *NOV (HS)* | + | 60350\|\|68244 |
| BICF2P188824 | 21485653 | T/A | B | *MAL2 (HS)* | + | -41525\|\|-30874 |
|  |  |  |  | *NOV (HS)* | + | 54928\|\|62822 |
| BICF2P823456 | 21512109 | G/A | BFA | *MAL2 (HS)* | + | -67981\|\|-57330 |
|  |  |  |  | *NOV (HS)* | + | 28472\|\|36366 |
| BICF2G630611822 | 21517868 | C/T | B | *MAL2 (HS)* | + | -73740\|\|-63089 |
|  |  |  |  | *NOV (HS)* | + | 22713\|\|30607 |
| BICF2G630611849 | 21544303 | T/C | BA | *NOV (HS)* | + | -3722\|\|4172 |
| BICF2P353630 | 21564309 | T/C | BA | *NOV (HS)* | + | -23728\|\|-15834 |
|  |  |  |  | *ENPP2 (HS)* | - | 136198\|\|55294 |
| BICF2P569328 | 21582897 | A/G | B | *NOV (HS)* | + | -42316\|\|-34422 |
|  |  |  |  | *ENPP2 (HS)* | - | 117610\|\|36706 |
| BICF2G630611925 | 21600902 | C/T | B | *NOV (HS)* | + | -60321\|\|-52427 |
|  |  |  |  | *ENPP2 (HS)* | - | 99605\|\|18701 |
| BICF2G630611947 | 21658633 | A/G | B | *ENPP2 (HS)* | - | 41874\|\|-39030 |
| BICF2G630611964 | 21690023 | C/T | BA | *ENPP2 (HS)* | - | 10484\|\|-70420 |
| BICF2G630611973 | 21701920 | G/A | B | *ENPP2 (HS)* | - | -1413\|\|-82317 |
|  |  |  |  | *TAF2 (HS)* | - | 156999\|\|73367 |
| BICF2G630611986 | 21735982 | T/G | B | *ENPP2 (HS)* | - | -35475\|\|-116379 |
|  |  |  |  | *TAF2 (HS)* | - | 122937\|\|39305 |
| BICF2G630612004 | 21761195 | A/G | B | *ENPP2 (HS)* | - | -60688\|\|-141592 |
|  |  |  |  | *TAF2 (HS)* | - | 97724\|\|14092 |
| BICF2G630612008 | 21787930 | C/G | B | *TAF2 (HS)* | - | 70989\|\|-12643 |
| BICF2G630612018 | 21860775 | A/G | B | *DSCC1 (HS)* | - | 15756\|\|-1390 |
| BICF2P315011 | 22006912 | C/T | B | *DEPTOR (HS)* | + | -112670\|\|17273 |
| BICF2G630612057 | 22012935 | C/T | B | *DEPTOR (HS)* | + | -118693\|\|11250 |
| BICF2S2304131 | 22051720 | C/T | BA | *DEPTOR (HS)* | + | -157478\|\|-27535 |
|  |  |  |  | *COL14A1 (HS)* | + | 33238\|\|232117 |
| BICF2P1159074 | 22104302 | G/A | B | *COL14A1 (HS)* | + | -19344\|\|179535 |
| BICF2G630612158 | 22222851 | C/T | B | *COL14A1 (HS)* | + | -137893\|\|60986 |
| BICF2P179778 | 22237963 | C/G | B | *COL14A1 (HS)* | + | -153005\|\|45874 |
| BICF2G630612204 | 22243767 | C/T | BA | *COL14A1 (HS)* | + | -158809\|\|40070 |
| BICF2S23726680 | 22326464 | T/C | B | *MRPL13 (HS)* | - | 22077\|\|-25345 |
| BICF2G630612284 | 22360484 | G/A | B | *MTBP (HS)* | + | -11915\|\|58900 |
| BICF2G630612396 | 22433373 | A/G | BA | *SNTB1* | - | 228636\|\|-4105 |
| BICF2G630612428 | 22467697 | G/A | BFA | *SNTB1* | - | 194312\|\|-38429 |
| BICF2G630612500 | 22518351 | A/G | BFA | *SNTB1* | - | 143658\|\|-89083 |
| BICF2G630612519 | 22540553 | C/A | B | *SNTB1* | - | 121456\|\|-111285 |
| BICF2G630612554 | 22570265 | A/G | B | *SNTB1* | - | 91744\|\|-140997 |
| BICF2G630612561 | 22574997 | A/G | BA | *SNTB1* | - | 87012\|\|-145729 |
| BICF2G630612583 | 22586357 | T/C | BA | *SNTB1* | - | 75652\|\|-157089 |
| BICF2S23259132 | 22666702 | C/A | BA | *SNTB1* | - | -4693\|\|-237434 |
|  |  |  |  | *HAS2 (HS)* | - | 711961\|\|681033 |
| BICF2G630612637 | 22683322 | C/T | BFA | *SNTB1* | - | -21313\|\|-254054 |
|  |  |  |  | *HAS2 (HS)* | - | 695341\|\|664413 |
| BICF2P1052982 | 22691061 | A/G | BA | *SNTB1* | - | -29052\|\|-261793 |
|  |  |  |  | *HAS2 (HS)* | - | 687602\|\|656674 |
| BICF2S23054517 | 22748362 | C/A | BFA | *SNTB1* | - | -86353\|\|-319094 |
|  |  |  |  | *HAS2 (HS)* | - | 630301\|\|599373 |
| BICF2G630612866 | 22854585 | C/T | BFA | *SNTB1* | - | -192576\|\|-425317 |
|  |  |  |  | *HAS2 (HS)* | - | 524078\|\|493150 |
| BICF2G630612923 | 22892752 | A/G | BFA | *SNTB1* | - | -230743\|\|-463484 |
|  |  |  |  | *HAS2 (HS)* | - | 485911\|\|454983 |
| BICF2S23353450 | 22902533 | T/C | BA | *SNTB1* | - | -240524\|\|-473265 |
|  |  |  |  | *HAS2 (HS)* | - | 476130\|\|445202 |
| BICF2S23121112 | 22937592 | A/C | BA | *SNTB1* | - | -275583\|\|-508324 |
|  |  |  |  | *HAS2 (HS)* | - | 441071\|\|410143 |
| BICF2G630613004 | 22959881 | T/C | BFA | *SNTB1* | - | -297872\|\|-530613 |
|  |  |  |  | *HAS2 (HS)* | - | 418782\|\|387854 |
| BICF2S2318454 | 22991514 | G/A | BA | *SNTB1* | - | -329505\|\|-562246 |
|  |  |  |  | *HAS2 (HS)* | - | 387149\|\|356221 |
| BICF2P240664 | 23004428 | C/T | BFA | *SNTB1* | - | -342419\|\|-575160 |
|  |  |  |  | *HAS2 (HS)* | - | 374235\|\|343307 |
| BICF2P648379 | 23023126 | A/G | BA | *SNTB1* | - | -361117\|\|-593858 |
|  |  |  |  | *HAS2 (HS)* | - | 355537\|\|324609 |
| BICF2S23444219 | 23039341 | A/G | BFA | *SNTB1* | - | -377332\|\|-610073 |
|  |  |  |  | *HAS2 (HS)* | - | 339322\|\|308394 |
| BICF2P1434455 | 23047599 | C/A | BFA | *SNTB1* | - | -385590\|\|-618331 |
|  |  |  |  | *HAS2 (HS)* | - | 331064\|\|300136 |
| BICF2G630613174 | 23180227 | C/A | B | *SNTB1* | - | -518218\|\|-750959 |
|  |  |  |  | *HAS2 (HS)* | - | 198436\|\|167508 |
| BICF2G630613246 | 23235401 | T/C | BA | *SNTB1* | - | -573392\|\|-806133 |
|  |  |  |  | *HAS2 (HS)* | - | 143262\|\|112334 |
| BICF2P942108 | 23430873 | T/C | BFAVH | *HAS2 (HS)* | - | -52210\|\|-83138 |
|  |  |  |  | *ZHX2 (HS)* | + | 838026\|\|999542 |
| BICF2G630613491 | 23487992 | C/T | BFAVH | *HAS2 (HS)* | - | -109329\|\|-140257 |
|  |  |  |  | *ZHX2 (HS)* | + | 780907\|\|942423 |
| BICF2G630613734 | 23680032 | C/T | BFAVH | *HAS2 (HS)* | - | -301369\|\|-332297 |
|  |  |  |  | *ZHX2 (HS)* | + | 588867\|\|750383 |
| BICF2G630613761 | 23706001 | G/C | BAVH | *HAS2 (HS)* | - | -327338\|\|-358266 |
|  |  |  |  | *ZHX2 (HS)* | + | 562898\|\|724414 |
| BICF2G630613823 | 23789037 | A/G | BFAVH | *HAS2 (HS)* | - | -410374\|\|-441302 |
|  |  |  |  | *ZHX2 (HS)* | + | 479862\|\|641378 |
| BICF2P257816 | 23827729 | T/C | BFAVH | *HAS2 (HS)* | - | -449066\|\|-479994 |
|  |  |  |  | *ZHX2 (HS)* | + | 441170\|\|602686 |
| BICF2P932753 | 23855195 | T/C | BFAVH | *HAS2 (HS)* | - | -476532\|\|-507460 |
|  |  |  |  | *ZHX2 (HS)* | + | 413704\|\|575220 |
| BICF2P932755 | 23855429 | G/A | BFAVH | *HAS2 (HS)* | - | -476766\|\|-507694 |
|  |  |  |  | *ZHX2 (HS)* | + | 413470\|\|574986 |
| BICF2S2294688 | 23893661 | C/T | BFAVH | *HAS2 (HS)* | - | -514998\|\|-545926 |
|  |  |  |  | *ZHX2 (HS)* | + | 375238\|\|536754 |
| BICF2S244264 | 23946082 | A/G | BFAVH | *HAS2 (HS)* | - | -567419\|\|-598347 |
|  |  |  |  | *ZHX2 (HS)* | + | 322817\|\|484333 |
| BICF2P1188454 | 24210601 | T/C | BAVH | *HAS2 (HS)* | - | -831938\|\|-862866 |
|  |  |  |  | *ZHX2 (HS)* | + | 58298\|\|219814 |
| BICF2P1318675 | 26440885 | G/C | A | *TRIB1 (HS)* | + | -64429\|\|-56160 |
|  |  |  |  | *FAM84B (HS)* | - | 814557\|\|810079 |
| BICF2G630616162 | 26584733 | A/G | FAVH | *TRIB1 (HS)* | + | -208277\|\|-200008 |
|  |  |  |  | *FAM84B (HS)* | - | 670709\|\|666231 |
| BICF2P1224625 | 26621147 | C/A | FAVH | *TRIB1 (HS)* | + | -244691\|\|-236422 |
|  |  |  |  | *FAM84B (HS)* | - | 634295\|\|629817 |
| BICF2P1207814 | 26717374 | A/G | FA | *TRIB1 (HS)* | + | -340918\|\|-332649 |
|  |  |  |  | *FAM84B (HS)* | - | 538068\|\|533590 |
| BICF2P1428283 | 26807341 | A/G | FA | *TRIB1 (HS)* | + | -430885\|\|-422616 |
|  |  |  |  | *FAM84B (HS)* | - | 448101\|\|443623 |
| BICF2S23157464 | 26854125 | G/A | FA | *TRIB1 (HS)* | + | -477669\|\|-469400 |
|  |  |  |  | *FAM84B (HS)* | - | 401317\|\|396839 |
| BICF2S23340302 | 26868889 | G/A | FA | *TRIB1 (HS)* | + | -492433\|\|-484164 |
|  |  |  |  | *FAM84B (HS)* | - | 386553\|\|382075 |
| BICF2G630616424 | 26911739 | G/A | FA | *TRIB1 (HS)* | + | -535283\|\|-527014 |
|  |  |  |  | *FAM84B (HS)* | - | 343703\|\|339225 |
| BICF2G630616465 | 26951845 | A/C | FA | *TRIB1 (HS)* | + | -575389\|\|-567120 |
|  |  |  |  | *FAM84B (HS)* | - | 303597\|\|299119 |
| BICF2G630616472 | 26959221 | A/G | FA | *TRIB1 (HS)* | + | -582765\|\|-574496 |
|  |  |  |  | *FAM84B (HS)* | - | 296221\|\|291743 |
| BICF2G630616516 | 27097125 | C/T | FA | *TRIB1 (HS)* | + | -720669\|\|-712400 |
|  |  |  |  | *FAM84B (HS)* | - | 158317\|\|153839 |
| BICF2G630616540 | 27120636 | G/A | FA | *TRIB1 (HS)* | + | -744180\|\|-735911 |
|  |  |  |  | *FAM84B (HS)* | - | 134806\|\|130328 |
| BICF2S23129171 | 27165724 | C/T | A | *TRIB1 (HS)* | + | -789268\|\|-780999 |
|  |  |  |  | *FAM84B (HS)* | - | 89718\|\|85240 |
| BICF2P915421 | 27190475 | C/T | FA | *TRIB1 (HS)* | + | -814019\|\|-805750 |
|  |  |  |  | *FAM84B (HS)* | - | 64967\|\|60489 |
| BICF2G630616697 | 27360384 | G/A | FA | *FAM84B (HS)* | - | -104942\|\|-109420 |
|  |  |  |  | *MYC (CF)* | + | 877624\|\|882161 |
| BICF2G630616702 | 27371905 | A/G | FA | *FAM84B (HS)* | - | -116463\|\|-120941 |
|  |  |  |  | *MYC (CF)* | + | 866103\|\|870640 |
| BICF2P1298613 | 27383736 | G/A | A | *FAM84B (HS)* | - | -128294\|\|-132772 |
|  |  |  |  | *MYC (CF)* | + | 854272\|\|858809 |
| BICF2P360830 | 27404911 | C/T | FA | *FAM84B (HS)* | - | -149469\|\|-153947 |
|  |  |  |  | *MYC (CF)* | + | 833097\|\|837634 |
| BICF2G630616806 | 27613738 | T/G | A | *FAM84B (HS)* | - | -358296\|\|-362774 |
|  |  |  |  | *MYC (CF)* | + | 624270\|\|628807 |
| BICF2P1396319 | 27663429 | A/G | FA | *FAM84B (HS)* | - | -407987\|\|-412465 |
|  |  |  |  | *MYC (CF)* | + | 574579\|\|579116 |
| BICF2G630616933 | 27752446 | A/G | A | *FAM84B (HS)* | - | -497004\|\|-501482 |
|  |  |  |  | *MYC (CF)* | + | 485562\|\|490099 |
| BICF2G630617043 | 27831739 | T/C | A | *FAM84B (HS)* | - | -576297\|\|-580775 |
|  |  |  |  | *MYC (CF)* | + | 406269\|\|410806 |
| BICF2P449473 | 27913803 | T/G | A | *FAM84B (HS)* | - | -658361\|\|-662839 |
|  |  |  |  | *MYC (CF)* | + | 324205\|\|328742 |
| BICF2G630617157 | 28006015 | C/T | FA | *FAM84B (HS)* | - | -750573\|\|-755051 |
|  |  |  |  | *MYC (CF)* | + | 231993\|\|236530 |
| BICF2P564681 | 28291984 | C/T | BA | *MYC (CF)* | + | -53976\|\|-49439 |
|  |  |  |  | *GSDMC (HS)* | + | 1643477\|\|1664262 |
| BICF2S23051140 | 28301987 | T/C | BA | *MYC (CF)* | + | -63979\|\|-59442 |
|  |  |  |  | *GSDMC (HS)* | + | 1633474\|\|1654259 |

^1^Phenotypic groups are breed subtype (B), fever (F), arthritis (A) and vesicular hyaluronosis (VH). ^2^Nearby gene is given as the canine version (CF) if annotated in *CanFam* 2.0 or the human orthologue (HS). ^3^Distance is given from transcription start to transcription stop based on gene orientation.
