# Supplementary material for: Thorough Investigation of a Canine Autoinflammatory Disease (AID) Confirms One Main Risk Locus and Suggests a Modifier Locus for Amyloidosis
Source: PLoS One. 2013 Oct 9;8(10):e75242. doi: 10.1371/journal.pone.0075242 (PMC3793984; doi:10.1371/journal.pone.0075242)
Supplement: Table S3 — Individuals used for chromosome 14 candidate gene study. (DOCX) [file pone.0075242.s005.docx]

**Table S3. Individuals used for chromosome 14 candidate gene study**

| Amyloidosis Status | Individual | Haplotype | |
| --- | --- | --- | --- |
| Negative | 248516 | H14-11 | TGGGGCCC/TGGGGCCC |
|  | 249295 | H14-11 | TGGGGCCC/TGGGGCCC |
|  | 257036 | H14-11 | TGGGGCCC/TGGGGCCC |
|  | 261948 | H14-110 | TGGGGCCC/CATCACCC |
|  | 266410 | H14-1111 | CATCATAT/CATCATAT |
|  | 270454 | N/A |  |
|  | 280243 | N/A |  |
| Positive | 266445 | H14-11 | TGGGGCCC/TGGGGCCC |
|  | 272699 | H14-11 | TGGGGCCC/TGGGGCCC |
|  | 244824 | H14-12 | TGGGGCCC/TGGGGCCT |
|  | 257055 | H14-12 | TGGGGCCC/TGGGGCCT |
|  | 266454 | H14-12 | TGGGGCCC/TGGGGCCT |
|  | 255160 | H14-111 | TGGGGCCC/CATCATAT |
|  | 170889 | H14-211 | TGGGGCCT/CATCATAT |

N/A: No DNA was available to genotype this individual
