# Supplementary material for: Thorough Investigation of a Canine Autoinflammatory Disease (AID) Confirms One Main Risk Locus and Suggests a Modifier Locus for Amyloidosis
Source: PLoS One. 2013 Oct 9;8(10):e75242. doi: 10.1371/journal.pone.0075242 (PMC3793984; doi:10.1371/journal.pone.0075242)
Supplement: Table S4 — Binary phenotypic description of each individual included in the study. (DOCX) [file pone.0075242.s006.docx]

**Table S4. Binary phenotypic description of each individual included in the study.**

|  |  |  | |  |  | | Health Control | | | | Shar-Pei Autoinflammatory Disease (SPAID) Symptom | | | | |
| --- | --- | --- | --- | --- | --- | --- | --- | --- | --- | --- | --- | --- | --- | --- | --- |
| Individual | Sex^1^ | Pop^2^ | Age^3^ | | | Breed Subtype^4^ | | Group 1 | Group 2 | Fever | | Arthritis | Vesicular Hyaluronosis | Otitis | Amyloid Status^5^ |
| 1 | 1 | 1 | 12 | | | NA | | Y | Y | NA | | NA | NA | NA | N |
| 2 | 1 | 1 | 12 | | | NA | | Y | Y | NA | | NA | NA | NA | N |
| 3 | 1 | 1 | 7 | | | BM | | Y | Y | NA | | NA | NA | NA | N |
| 4 | 1 | 1 | 12 | | | NA | | NA | NA | NA | | NA | NA | NA | N |
| 5 | 1 | 4 | 7 | | | NA | | NA | NA | Y | | Y | NA | Y | N |
| 6 | 1 | 1 | 5 | | | NA | | NA | NA | NA | | NA | NA | NA | N |
| 7 | 2 | 1 | 8 | | | NA | | Y | Y | NA | | NA | NA | NA | N |
| 8 | 2 | 1 | 6 | | | MM | | NA | Y | NA | | NA | NA | NA | N |
| 9 | 2 | 1 | 6 | | | NA | | NA | Y | NA | | NA | NA | NA | N |
| 10 | 2 | 1 | 5 | | | NA | | NA | Y | NA | | NA | NA | NA | N |
| 11 | 2 | 1 | 6 | | | NA | | NA | NA | NA | | NA | NA | NA | N |
| 12 | 2 | 1 | 5 | | | NA | | NA | NA | NA | | NA | NA | NA | N |
| 13 | 2 | 1 | 4 | | | NA | | NA | NA | NA | | NA | NA | NA | N |
| 14 | 2 | 1 | 3 | | | NA | | NA | NA | NA | | NA | NA | NA | N |
| 15 | 1 | 2 | NA | | | MM | | NA | NA | Y | | Y | Y | Y | Y |
| 16 | 1 | 3 | 6 | | | NA | | NA | NA | NA | | NA | NA | NA | Y |
| 17 | 1 | 4 | NA | | | NA | | NA | NA | Y | | NA | NA | NA | Y |
| 18 | 1 | 4 | NA | | | NA | | NA | NA | Y | | NA | NA | NA | Y |
| 19 | 1 | 4 | NA | | | NA | | NA | NA | NA | | Y | NA | NA | Y |
| 20 | 1 | 4 | NA | | | NA | | NA | NA | NA | | Y | NA | NA | Y |
| 21 | 1 | 4 | NA | | | NA | | NA | NA | Y | | NA | NA | NA | Y |
| 22 | 1 | 4 | NA | | | NA | | NA | NA | NA | | Y | NA | NA | Y |
| 23 | 1 | 1 | 10 | | | NA | | NA | NA | NA | | NA | NA | NA | Y |
| 24 | 1 | 4 | 10 | | | MM | | NA | NA | NA | | NA | NA | NA | Y |
| 25 | 1 | 1 | 7 | | | MM | | NA | NA | NA | | Y | NA | NA | Y |
| 26 | 1 | 1 | 6 | | | MM | | NA | NA | Y | | NA | NA | NA | Y |
| 27 | 1 | 3 | 5 | | | MM | | NA | NA | NA | | NA | NA | NA | Y |
| 28 | 1 | 4 | 5 | | | MM | | NA | NA | Y | | Y | Y | NA | Y |
| 29 | 1 | 3 | 4 | | | NA | | NA | NA | NA | | NA | NA | Y | Y |
| 30 | 1 | 4 | 4 | | | MM | | NA | NA | Y | | Y | Y | Y | Y |
| 31 | 1 | 1 | 2 | | | NA | | NA | NA | Y | | NA | NA | NA | Y |
| 32 | 2 | 2 | NA | | | MM | | NA | NA | NA | | Y | NA | NA | Y |
| 33 | 2 | 2 | NA | | | BM | | NA | NA | Y | | Y | NA | NA | Y |
| 34 | 2 | 3 | 5 | | | NA | | NA | NA | NA | | Y | NA | NA | Y |
| 35 | 2 | 4 | NA | | | NA | | NA | NA | Y | | NA | NA | NA | Y |
| 36 | 2 | 4 | NA | | | NA | | NA | NA | Y | | NA | NA | NA | Y |
| 37 | 2 | 4 | NA | | | NA | | NA | NA | Y | | NA | NA | NA | Y |
| 38 | 2 | 4 | NA | | | NA | | NA | NA | Y | | NA | NA | NA | Y |
| 39 | 2 | 4 | NA | | | NA | | NA | NA | NA | | Y | NA | NA | Y |
| 40 | 2 | 4 | 13 | | | MM | | NA | NA | NA | | NA | NA | NA | Y |
| 41 | 2 | 1 | 9 | | | MM | | NA | NA | NA | | NA | NA | NA | Y |
| 42 | 2 | 4 | 9 | | | MM | | NA | NA | Y | | NA | NA | NA | Y |
| 43 | 2 | 1 | 8 | | | MM | | NA | NA | NA | | NA | NA | NA | Y |
| 44 | 2 | 2 | 7 | | | BM | | NA | NA | NA | | NA | NA | NA | Y |
| 45 | 2 | 2 | 7 | | | MM | | NA | NA | Y | | NA | NA | NA | Y |
| 46 | 2 | 4 | 7 | | | NA | | NA | NA | Y | | Y | Y | NA | Y |
| 47 | 2 | 1 | 5 | | | NA | | NA | NA | NA | | NA | NA | NA | Y |
| 48 | 2 | 1 | 3 | | | BM | | NA | NA | NA | | NA | NA | NA | Y |
| 49 | 2 | 3 | 3 | | | MM | | NA | NA | Y | | Y | NA | Y | Y |
| 50 | 2 | 3 | 3 | | | NA | | NA | NA | Y | | Y | NA | Y | Y |
| 51 | 2 | 3 | 2 | | | MM | | NA | NA | NA | | NA | NA | NA | Y |
| 52 | 1 | 4 | 14 | | | BM | | Y | Y | NA | | NA | NA | NA | NA |
| 53 | 1 | 2 | 12 | | | MM | | Y | Y | NA | | NA | NA | NA | NA |
| 54 | 1 | 4 | 11 | | | NA | | Y | Y | NA | | NA | NA | NA | NA |
| 55 | 1 | 4 | 10 | | | BM | | Y | Y | NA | | NA | NA | NA | NA |
| 56 | 1 | 4 | 10 | | | NA | | Y | Y | NA | | NA | NA | NA | NA |
| 57 | 1 | 4 | 10 | | | MM | | Y | Y | NA | | NA | NA | NA | NA |
| 58 | 1 | 4 | 10 | | | NA | | Y | Y | NA | | NA | NA | NA | NA |
| 59 | 1 | 4 | 9 | | | NA | | Y | Y | NA | | NA | NA | NA | NA |
| 60 | 1 | 4 | 9 | | | BM | | Y | Y | NA | | NA | NA | NA | NA |
| 61 | 1 | 4 | 9 | | | BM | | Y | Y | NA | | NA | NA | NA | NA |
| 62 | 1 | 3 | 8 | | | BM | | Y | Y | NA | | NA | NA | NA | NA |
| 63 | 1 | 4 | 8 | | | NA | | Y | Y | NA | | NA | NA | NA | NA |
| 64 | 1 | 4 | 8 | | | NA | | Y | Y | NA | | NA | NA | NA | NA |
| 65 | 1 | 3 | 7 | | | BM | | Y | Y | NA | | NA | NA | NA | NA |
| 66 | 1 | 4 | 7 | | | NA | | NA | Y | NA | | NA | NA | NA | NA |
| 67 | 1 | 4 | 6 | | | MM | | NA | Y | NA | | NA | NA | NA | NA |
| 68 | 1 | 4 | 6 | | | MM | | NA | Y | NA | | NA | NA | NA | NA |
| 69 | 1 | 1 | 5 | | | NA | | NA | Y | NA | | NA | NA | NA | NA |
| 70 | 1 | 2 | 5 | | | BM | | NA | Y | NA | | NA | NA | NA | NA |
| 71 | 1 | 4 | 5 | | | NA | | NA | Y | NA | | NA | NA | NA | NA |
| 72 | 1 | 4 | 5 | | | NA | | NA | Y | NA | | NA | NA | NA | NA |
| 73 | 1 | 4 | 5 | | | MM | | NA | Y | NA | | NA | NA | NA | NA |
| 74 | 1 | 2 | NA | | | MM | | NA | NA | NA | | NA | Y | NA | NA |
| 75 | 1 | 2 | NA | | | BM | | NA | NA | Y | | Y | NA | NA | NA |
| 76 | 1 | 2 | NA | | | MM | | NA | NA | NA | | NA | Y | Y | NA |
| 77 | 1 | 2 | NA | | | MM | | NA | NA | NA | | NA | NA | NA | NA |
| 78 | 1 | 2 | NA | | | MM | | NA | NA | NA | | NA | NA | NA | NA |
| 79 | 1 | 2 | NA | | | MM | | NA | NA | NA | | NA | NA | NA | NA |
| 80 | 1 | 2 | NA | | | MM | | NA | NA | NA | | NA | NA | NA | NA |
| 81 | 1 | 2 | NA | | | MM | | NA | NA | NA | | NA | NA | NA | NA |
| 82 | 1 | 2 | NA | | | MM | | NA | NA | NA | | NA | NA | NA | NA |
| 83 | 1 | 2 | NA | | | MM | | NA | NA | NA | | NA | NA | NA | NA |
| 84 | 1 | 2 | NA | | | MM | | NA | NA | NA | | NA | NA | NA | NA |
| 85 | 1 | 2 | NA | | | NA | | NA | NA | NA | | NA | NA | NA | NA |
| 86 | 1 | 2 | NA | | | MM | | NA | NA | NA | | NA | NA | NA | NA |
| 87 | 1 | 3 | 7 | | | NA | | NA | NA | Y | | Y | NA | NA | NA |
| 88 | 1 | 4 | NA | | | NA | | NA | NA | Y | | NA | NA | NA | NA |
| 89 | 1 | 4 | 13 | | | MM | | NA | NA | Y | | Y | NA | NA | NA |
| 90 | 1 | 2 | 12 | | | BM | | NA | NA | Y | | Y | NA | NA | NA |
| 91 | 1 | 4 | 11 | | | NA | | NA | NA | Y | | Y | NA | NA | NA |
| 92 | 1 | 2 | 10 | | | NA | | NA | NA | Y | | Y | NA | NA | NA |
| 93 | 1 | 2 | 10 | | | MM | | NA | NA | Y | | Y | NA | NA | NA |
| 94 | 1 | 3 | 9 | | | NA | | NA | NA | Y | | NA | NA | NA | NA |
| 95 | 1 | 3 | 8 | | | MM | | NA | NA | Y | | Y | NA | NA | NA |
| 96 | 1 | 1 | 7 | | | NA | | NA | NA | Y | | Y | NA | NA | NA |
| 97 | 1 | 2 | 7 | | | NA | | NA | NA | Y | | NA | NA | NA | NA |
| 98 | 1 | 4 | 7 | | | MM | | NA | NA | Y | | Y | Y | NA | NA |
| 99 | 1 | 4 | 7 | | | NA | | NA | NA | Y | | NA | NA | NA | NA |
| 100 | 1 | 4 | 7 | | | MM | | NA | NA | Y | | Y | Y | NA | NA |
| 101 | 1 | 4 | 7 | | | NA | | NA | NA | Y | | Y | Y | NA | NA |
| 102 | 1 | 4 | 7 | | | NA | | NA | NA | NA | | NA | NA | Y | NA |
| 103 | 1 | 2 | 6 | | | NA | | NA | NA | Y | | Y | NA | NA | NA |
| 104 | 1 | 3 | 6 | | | MM | | NA | NA | Y | | Y | NA | NA | NA |
| 105 | 1 | 4 | 6 | | | MM | | NA | NA | Y | | NA | NA | NA | NA |
| 106 | 1 | 4 | 6 | | | NA | | NA | NA | Y | | Y | Y | NA | NA |
| 107 | 1 | 4 | 6 | | | NA | | NA | NA | Y | | Y | Y | NA | NA |
| 108 | 1 | 4 | 6 | | | MM | | NA | NA | NA | | NA | NA | Y | NA |
| 109 | 1 | 4 | 6 | | | NA | | NA | NA | Y | | Y | NA | Y | NA |
| 110 | 1 | 4 | 6 | | | MM | | NA | NA | Y | | Y | NA | Y | NA |
| 111 | 1 | 4 | 6 | | | MM | | NA | NA | NA | | NA | Y | NA | NA |
| 112 | 1 | 4 | 6 | | | MM | | NA | NA | Y | | NA | NA | NA | NA |
| 113 | 1 | 2 | 5 | | | BM | | NA | NA | Y | | Y | NA | NA | NA |
| 114 | 1 | 2 | 5 | | | MM | | NA | NA | Y | | Y | NA | NA | NA |
| 115 | 1 | 2 | 5 | | | BM | | NA | NA | Y | | Y | NA | NA | NA |
| 116 | 1 | 2 | 5 | | | BM | | NA | NA | Y | | Y | NA | NA | NA |
| 117 | 1 | 3 | 5 | | | NA | | NA | NA | Y | | Y | NA | NA | NA |
| 118 | 1 | 3 | 5 | | | MM | | NA | NA | Y | | Y | NA | NA | NA |
| 119 | 1 | 4 | 5 | | | BM | | NA | NA | NA | | NA | Y | NA | NA |
| 120 | 1 | 4 | 5 | | | MM | | NA | NA | Y | | Y | Y | Y | NA |
| 121 | 1 | 4 | 5 | | | MM | | NA | NA | Y | | Y | Y | Y | NA |
| 122 | 1 | 1 | 4 | | | NA | | NA | NA | NA | | NA | NA | Y | NA |
| 123 | 1 | 3 | 4 | | | NA | | NA | NA | Y | | NA | NA | NA | NA |
| 124 | 1 | 4 | 4 | | | MM | | NA | NA | Y | | Y | NA | NA | NA |
| 125 | 1 | 4 | 4 | | | MM | | NA | NA | Y | | Y | NA | NA | NA |
| 126 | 1 | 4 | 4 | | | NA | | NA | NA | NA | | NA | NA | NA | NA |
| 127 | 1 | 4 | 4 | | | NA | | NA | NA | NA | | NA | NA | NA | NA |
| 128 | 1 | 4 | 4 | | | MM | | NA | NA | NA | | NA | NA | NA | NA |
| 129 | 1 | 2 | 3 | | | MM | | NA | NA | Y | | NA | NA | NA | NA |
| 130 | 1 | 3 | 3 | | | MM | | NA | NA | Y | | Y | NA | NA | NA |
| 131 | 1 | 4 | 3 | | | NA | | NA | NA | Y | | NA | NA | NA | NA |
| 132 | 1 | 4 | 3 | | | MM | | NA | NA | Y | | Y | NA | NA | NA |
| 133 | 1 | 4 | 3 | | | NA | | NA | NA | Y | | Y | NA | NA | NA |
| 134 | 1 | 4 | 3 | | | NA | | NA | NA | NA | | Y | Y | Y | NA |
| 135 | 1 | 4 | 3 | | | MM | | NA | NA | Y | | NA | Y | NA | NA |
| 136 | 1 | 4 | 3 | | | MM | | NA | NA | Y | | NA | NA | NA | NA |
| 137 | 1 | 4 | 3 | | | MM | | NA | NA | NA | | NA | NA | NA | NA |
| 138 | 1 | 4 | 3 | | | NA | | NA | NA | NA | | NA | NA | NA | NA |
| 139 | 1 | 4 | 3 | | | MM | | NA | NA | NA | | NA | NA | NA | NA |
| 140 | 1 | 4 | 3 | | | MM | | NA | NA | NA | | NA | NA | NA | NA |
| 141 | 1 | 2 | 2 | | | MM | | NA | NA | Y | | NA | NA | NA | NA |
| 142 | 1 | 2 | 2 | | | MM | | NA | NA | Y | | Y | Y | NA | NA |
| 143 | 1 | 4 | 2 | | | NA | | NA | NA | NA | | Y | NA | NA | NA |
| 144 | 1 | 4 | 2 | | | NA | | NA | NA | Y | | Y | NA | Y | NA |
| 145 | 1 | 4 | 2 | | | NA | | NA | NA | Y | | Y | Y | NA | NA |
| 146 | 1 | 4 | 2 | | | NA | | NA | NA | Y | | NA | NA | NA | NA |
| 147 | 1 | 4 | 2 | | | MM | | NA | NA | Y | | Y | Y | Y | NA |
| 148 | 1 | 4 | 2 | | | MM | | NA | NA | Y | | NA | NA | Y | NA |
| 149 | 1 | 4 | 2 | | | MM | | NA | NA | Y | | NA | NA | NA | NA |
| 150 | 1 | 4 | 2 | | | MM | | NA | NA | NA | | NA | NA | NA | NA |
| 151 | 1 | 2 | 1 | | | MM | | NA | NA | Y | | Y | NA | NA | NA |
| 152 | 1 | 4 | 1 | | | NA | | NA | NA | Y | | Y | NA | NA | NA |
| 153 | 1 | 4 | 1 | | | MM | | NA | NA | Y | | Y | NA | NA | NA |
| 154 | 1 | 4 | 1 | | | NA | | NA | NA | Y | | Y | NA | NA | NA |
| 155 | 1 | 4 | 0 | | | BM | | NA | NA | Y | | NA | NA | NA | NA |
| 156 | 1 | 4 | 0 | | | MM | | NA | NA | Y | | Y | NA | NA | NA |
| 157 | 2 | 4 | 13 | | | MM | | Y | Y | NA | | NA | NA | NA | NA |
| 158 | 2 | 2 | 11 | | | MM | | Y | Y | NA | | NA | NA | NA | NA |
| 159 | 2 | 4 | 11 | | | BM | | Y | Y | NA | | NA | NA | NA | NA |
| 160 | 2 | 4 | 10 | | | NA | | Y | Y | NA | | NA | NA | NA | NA |
| 161 | 2 | 4 | 8 | | | NA | | Y | Y | NA | | NA | NA | NA | NA |
| 162 | 2 | 4 | 7 | | | NA | | Y | Y | NA | | NA | NA | NA | NA |
| 163 | 2 | 4 | 6 | | | BM | | NA | Y | NA | | NA | NA | NA | NA |
| 164 | 2 | 1 | 5 | | | MM | | NA | NA | Y | | Y | NA | NA | NA |
| 165 | 2 | 1 | 5 | | | NA | | NA | NA | Y | | NA | NA | NA | NA |
| 166 | 2 | 2 | NA | | | MM | | NA | NA | Y | | Y | Y | NA | NA |
| 167 | 2 | 2 | NA | | | NA | | NA | NA | Y | | Y | Y | NA | NA |
| 168 | 2 | 2 | NA | | | MM | | NA | NA | Y | | Y | NA | NA | NA |
| 169 | 2 | 2 | NA | | | MM | | NA | NA | Y | | Y | Y | NA | NA |
| 170 | 2 | 2 | NA | | | MM | | NA | NA | Y | | Y | Y | NA | NA |
| 171 | 2 | 2 | NA | | | NA | | NA | NA | NA | | NA | Y | NA | NA |
| 172 | 2 | 2 | NA | | | NA | | NA | NA | NA | | NA | Y | NA | NA |
| 173 | 2 | 2 | NA | | | BM | | NA | NA | Y | | NA | NA | NA | NA |
| 174 | 2 | 2 | NA | | | MM | | NA | NA | NA | | NA | NA | NA | NA |
| 175 | 2 | 2 | NA | | | MM | | NA | NA | NA | | NA | NA | NA | NA |
| 176 | 2 | 2 | NA | | | MM | | NA | NA | NA | | NA | NA | NA | NA |
| 177 | 2 | 2 | NA | | | MM | | NA | NA | NA | | NA | NA | NA | NA |
| 178 | 2 | 2 | NA | | | MM | | NA | NA | NA | | NA | NA | NA | NA |
| 179 | 2 | 2 | NA | | | BM | | NA | NA | NA | | NA | NA | NA | NA |
| 180 | 2 | 2 | NA | | | BM | | NA | NA | NA | | NA | NA | NA | NA |
| 181 | 2 | 2 | NA | | | BM | | NA | NA | NA | | NA | NA | NA | NA |
| 182 | 2 | 2 | NA | | | BM | | NA | NA | NA | | NA | NA | NA | NA |
| 183 | 2 | 2 | NA | | | NA | | NA | NA | NA | | NA | NA | NA | NA |
| 184 | 2 | 2 | NA | | | MM | | NA | NA | NA | | NA | NA | NA | NA |
| 185 | 2 | 2 | NA | | | MM | | NA | NA | NA | | NA | NA | NA | NA |
| 186 | 2 | 2 | NA | | | NA | | NA | NA | NA | | NA | NA | NA | NA |
| 187 | 2 | 2 | NA | | | MM | | NA | NA | NA | | NA | NA | NA | NA |
| 188 | 2 | 3 | 5 | | | NA | | NA | NA | NA | | NA | Y | Y | NA |
| 189 | 2 | 4 | NA | | | NA | | NA | NA | Y | | NA | NA | NA | NA |
| 190 | 2 | 4 | NA | | | MM | | NA | NA | Y | | NA | NA | NA | NA |
| 191 | 2 | 4 | NA | | | BM | | NA | NA | Y | | Y | NA | NA | NA |
| 192 | 2 | 4 | NA | | | NA | | NA | NA | NA | | NA | Y | NA | NA |
| 193 | 2 | 4 | NA | | | NA | | NA | NA | NA | | NA | NA | NA | NA |
| 194 | 2 | 4 | NA | | | NA | | NA | NA | NA | | NA | NA | NA | NA |
| 195 | 2 | 4 | 12 | | | NA | | NA | NA | Y | | NA | NA | NA | NA |
| 196 | 2 | 2 | 9 | | | MM | | NA | NA | Y | | Y | NA | NA | NA |
| 197 | 2 | 4 | 9 | | | MM | | NA | NA | Y | | Y | Y | NA | NA |
| 198 | 2 | 2 | 8 | | | BM | | NA | NA | Y | | Y | NA | NA | NA |
| 199 | 2 | 4 | 8 | | | MM | | NA | NA | NA | | NA | Y | NA | NA |
| 200 | 2 | 2 | 7 | | | MM | | NA | NA | Y | | Y | Y | NA | NA |
| 201 | 2 | 2 | 7 | | | BM | | NA | NA | Y | | Y | NA | NA | NA |
| 202 | 2 | 2 | 7 | | | NA | | NA | NA | Y | | Y | NA | NA | NA |
| 203 | 2 | 4 | 7 | | | BM | | NA | NA | Y | | Y | Y | NA | NA |
| 204 | 2 | 4 | 7 | | | MM | | NA | NA | Y | | NA | NA | NA | NA |
| 205 | 2 | 4 | 7 | | | NA | | NA | NA | Y | | Y | Y | NA | NA |
| 206 | 2 | 4 | 7 | | | MM | | NA | NA | NA | | NA | NA | Y | NA |
| 207 | 2 | 1 | 6 | | | NA | | NA | NA | Y | | Y | NA | NA | NA |
| 208 | 2 | 2 | 6 | | | BM | | NA | NA | Y | | Y | NA | NA | NA |
| 209 | 2 | 2 | 6 | | | MM | | NA | NA | NA | | Y | Y | NA | NA |
| 210 | 2 | 2 | 6 | | | MM | | NA | NA | NA | | Y | NA | NA | NA |
| 211 | 2 | 2 | 6 | | | MM | | NA | NA | NA | | Y | NA | NA | NA |
| 212 | 2 | 3 | 6 | | | MM | | NA | NA | Y | | Y | NA | NA | NA |
| 213 | 2 | 4 | 6 | | | BM | | NA | NA | Y | | Y | Y | NA | NA |
| 214 | 2 | 4 | 6 | | | MM | | NA | NA | NA | | Y | Y | NA | NA |
| 215 | 2 | 4 | 6 | | | MM | | NA | NA | Y | | Y | Y | NA | NA |
| 216 | 2 | 4 | 6 | | | MM | | NA | NA | Y | | Y | Y | NA | NA |
| 217 | 2 | 1 | 5 | | | MM | | NA | NA | Y | | Y | NA | NA | NA |
| 218 | 2 | 2 | 5 | | | MM | | NA | NA | Y | | Y | NA | NA | NA |
| 219 | 2 | 2 | 5 | | | MM | | NA | NA | Y | | Y | Y | NA | NA |
| 220 | 2 | 4 | 5 | | | MM | | NA | NA | Y | | Y | NA | Y | NA |
| 221 | 2 | 4 | 5 | | | MM | | NA | NA | Y | | Y | Y | NA | NA |
| 222 | 2 | 3 | 4 | | | BM | | NA | NA | NA | | NA | NA | Y | NA |
| 223 | 2 | 4 | 4 | | | MM | | NA | NA | Y | | Y | Y | NA | NA |
| 224 | 2 | 4 | 4 | | | NA | | NA | NA | Y | | Y | NA | NA | NA |
| 225 | 2 | 4 | 4 | | | MM | | NA | NA | Y | | Y | NA | NA | NA |
| 226 | 2 | 4 | 4 | | | MM | | NA | NA | NA | | NA | Y | NA | NA |
| 227 | 2 | 4 | 4 | | | MM | | NA | NA | Y | | Y | NA | NA | NA |
| 228 | 2 | 4 | 4 | | | MM | | NA | NA | Y | | NA | NA | NA | NA |
| 229 | 2 | 4 | 4 | | | MM | | NA | NA | Y | | Y | NA | NA | NA |
| 230 | 2 | 4 | 4 | | | NA | | NA | NA | NA | | NA | NA | NA | NA |
| 231 | 2 | 4 | 4 | | | MM | | NA | NA | NA | | NA | NA | NA | NA |
| 232 | 2 | 4 | 3 | | | NA | | NA | NA | Y | | Y | NA | NA | NA |
| 233 | 2 | 4 | 3 | | | MM | | NA | NA | Y | | Y | Y | NA | NA |
| 234 | 2 | 4 | 3 | | | NA | | NA | NA | Y | | Y | NA | NA | NA |
| 235 | 2 | 4 | 3 | | | MM | | NA | NA | Y | | NA | NA | NA | NA |
| 236 | 2 | 4 | 3 | | | MM | | NA | NA | Y | | Y | NA | NA | NA |
| 237 | 2 | 4 | 3 | | | MM | | NA | NA | NA | | NA | NA | NA | NA |
| 238 | 2 | 4 | 3 | | | NA | | NA | NA | NA | | NA | NA | Y | NA |
| 239 | 2 | 4 | 3 | | | MM | | NA | NA | NA | | NA | NA | NA | NA |
| 240 | 2 | 4 | 3 | | | MM | | NA | NA | NA | | NA | NA | NA | NA |
| 241 | 2 | 4 | 3 | | | MM | | NA | NA | NA | | NA | NA | NA | NA |
| 242 | 2 | 1 | 2 | | | NA | | NA | NA | Y | | NA | NA | NA | NA |
| 243 | 2 | 2 | 2 | | | MM | | NA | NA | Y | | Y | NA | NA | NA |
| 244 | 2 | 2 | 2 | | | NA | | NA | NA | Y | | Y | NA | NA | NA |
| 245 | 2 | 4 | 2 | | | MM | | NA | NA | NA | | Y | Y | Y | NA |
| 246 | 2 | 4 | 2 | | | NA | | NA | NA | Y | | Y | NA | NA | NA |
| 247 | 2 | 4 | 2 | | | MM | | NA | NA | NA | | Y | Y | NA | NA |
| 248 | 2 | 4 | 2 | | | MM | | NA | NA | Y | | Y | NA | Y | NA |
| 249 | 2 | 4 | 2 | | | MM | | NA | NA | Y | | NA | NA | NA | NA |
| 250 | 2 | 4 | 2 | | | MM | | NA | NA | Y | | Y | NA | Y | NA |
| 251 | 2 | 2 | 1 | | | BM | | NA | NA | Y | | Y | NA | NA | NA |
| 252 | 2 | 4 | 1 | | | NA | | NA | NA | Y | | Y | NA | NA | NA |
| 253 | 2 | 4 | 1 | | | NA | | NA | NA | Y | | NA | NA | NA | NA |
| 254 | 2 | 4 | 1 | | | MM | | NA | NA | Y | | Y | Y | Y | NA |
| 255 | 2 | 4 | 0 | | | MM | | NA | NA | NA | | Y | NA | NA | NA |

Membership of an individual to a group is indicated as yes (Y) or not applicable (NA). ^1^Sex is designated as male (1), female (2). ^2^The geographic origin of each individual is coded as France (1), the Netherlands (2), Sweden (3) or the United States of America (4). ^3^Age of disease diagnosis or current age if still healthy is indicated in years. ^4^Breed subtype is designated for meatmouth (MM) and bonemouth (BM) individuals. ^5^The amyloidosis renal biopsy diagnosis in given as positive (Y), negative (N) or not tested (NA).
