# Supplementary material for: Thorough Investigation of a Canine Autoinflammatory Disease (AID) Confirms One Main Risk Locus and Suggests a Modifier Locus for Amyloidosis
Source: PLoS One. 2013 Oct 9;8(10):e75242. doi: 10.1371/journal.pone.0075242 (PMC3793984; doi:10.1371/journal.pone.0075242)
Supplement: Table S5 — Primer list for candidate gene expression. (DOCX) [file pone.0075242.s007.docx]

**Table S5. Primer list for candidate gene expression.**

| Gene | Primer | Sequence 5'-3' | Chromosome | Strand | Start (bp) | Stop (bp) |
| --- | --- | --- | --- | --- | --- | --- |
| *DNAH11* | DNAH11-RT-F | TTCCTGACAGTGATGTCCAAC | 14 | + | 38734192 | 38734212 |
|  | DNAH11-RT-R | TCTTCTGGGAGTTTCTCCAAA | 14 | - | 38736972 | 38736992 |
| *RAPGEF5* | RAPGEF5-RT-F2 | CAGCTGTGGGTCCTCACTAA | 14 | + | 38941479 | 38941498 |
|  | RAPGEF5-RT-R2 | CTCAAAGCAGCAGCTCATGT | 14 | - | 38941603 | 38941622 |
| *IL6* | IL6-RT-F2 | AAGACAGCAAAGAGGCACTG | 14 | + | 39434398 | 39434417 |
|  | IL6-RT-R2 | GAAACTCCACAAGACCGGTAG | 14 | - | 39435206 | 39435226 |
| *IGF2BP3* | IGF2BP3-RT-F | CTGGTCACGAGGGACAACTA | 14 | + | 39930363 | 39930382 |
|  | IGF2BP3-RT-R | CTGCTGGCAGAGTTATTGGA | 14 | - | 39931117 | 39931136 |
| *TAX1BP1* | TAX1BP1-RT-F | AGGAAATTCTGACATGTTGGTG | 14 | + | 43825807 | 43825828 |
|  | TAX1BP1-RT-R | TTGTTCTCGAAGTTGTGTTGTTT | 14 | - | 43829597 | 43829619 |
| *TRIL* | TRIL-RT-F | TGCCATTGCTCTACACATGA | 14 | + | 44955551 | 44955570 |
|  | TRIL-RT-R | GGGTCGCAGCTCATTCTTAT | 14 | - | 44955619 | 44955638 |
| *SCRN1* | SCRN1-RT-F | CTTTCTCAGCTTGCGACCTT | 14 | + | 45680463 | 45680482 |
|  | SCRN1-RT-R | AGCACAGTCTCCTTGCTTTG | 14 | - | 45682663 | 45682682 |
| *NOD1* | NOD1-RT-F | CTTTGCACTCATCCAGGATTC | 14 | + | 46146245 | 46146265 |
|  | NOD1-RT-R | GCGTAAACCAGATCACTGACA | 14 | - | 46148026 | 46148046 |
| *GARS* | GARS-RT-F | TTAATGTTCAAGACCTTCATTGG | 14 | + | 46282053 | 46282075 |
|  | GARS-RT-R | ATTTCCAATCTGAGCAGCAG | 14 | - | 46284342 | 46284361 |
| *CRHR2* | CRHR2-RT-F2 | GGGTTGACGAAGAAGAGCAT | 14 | + | 46325873 | 46325892 |
|  | CRHR2-RT-R2 | CATCCACCACCTCAGAGACA | 14 | - | 46326137 | 46326156 |
| *AQP1* | AQP1-RT-F | GCCATGATCCTCTTCGTCTT | 14 | + | 46547716 | 46547735 |
|  | AQP1-RT-R | CACCTGCTGTCTGGTTGTTC | 14 | - | 46547778 | 46547797 |
| *NT5C3* | NT5C3-RT-F | TCTTCAAGGCACCATCATGT | 14 | + | 48038871 | 48038890 |
|  | NT5C3-RT-R | GAGGAGGTGATCCATCAAGC | 14 | - | 48039706 | 48039725 |
| *SEPT7* | SEPT7-RT-F | GTGTTCAGTTGCTGCTCACA | 14 | + | 50423103 | 50423122 |
|  | SEPT7-RT-R | AGGCATCTGACGTCTGTTCA | 14 | - | 50424349 | 50424368 |
| *AOAH* | AOAH-RT-F | TCGAAGTTCGTAAATTTCTTGC | 14 | + | 50871716 | 50871737 |
|  | AOAH-RT-R | TGGATGTCCTCCAACAAGAC | 14 | - | 50873088 | 50873107 |
| *ELMO1* | ELMO1-RT-F | TCCAGGATTTCTGGTTGAATC | 14 | + | 51158443 | 51158463 |
|  | ELMO1-RT-R | CCGTCAGTCTGAGAGGATGA | 14 | - | 51166427 | 51166446 |
| *DNAJB9* | DNAJB9-RT-F2 | GTGTTTGGTCCCTGGCTAAT | 14 | + | 51846554 | 51846573 |
|  | DNAJB9-RT-R2 | GAGTGCAAAGAGGAGGAACC | 14 | - | 51846682 | 51846701 |
| *ZNF277* | ZNF277-F | CACCACCGCTTTAGAAGGTT | 14 | + | 54879191 | 54879210 |
|  | ZNF277-R | GCAACCAACTTGACATCAGC | 14 | - | 54879309 | 54879328 |
| *IFRD1* | IFRD1-RT-F | TTCATTGACTTGACCCTGGA | 14 | + | 55019256 | 55019275 |
|  | IFRD1-RT-R | GCTCAATGCTGTCTGTCAAAG | 14 | - | 55020078 | 55020098 |
| *FOXP2* | FOXP2-F3 | CTGAGGCACGGTTATTGCTA | 14 | + | 56364867 | 56364886 |
|  | FOXP2-R3 | TCATATGCCAAGAACCAACC | 14 | - | 56364924 | 56364943 |
| *HAS2* | HAS2-RT-F1 | TGTACATTCCCAGAGGTCCA | 13 | + | 23349570 | 23349589 |
|  | HAS2-RT-R1 | AGTCATGTACACGGCCTTCA | 13 | - | 23364318 | 23364337 |
| *MYC* | MYC-F | GAGACATGGTGAACCAGAGC | 13 | + | 28240425 | 28240444 |
|  | MYC-R | TTGATGAAGGTCTCGTCGTC | 13 | - | 28240460 | 28240479 |
| House Keeper |  |  |  |  |  |  |
| *EEF2* | EEF2-RT-F | GTATGTGGCCAAATTTGCTG | 20 | + | 58526371 | 58526390 |
|  | EEF2-RT-R | TGGCTGACTTGCTGAATTTG | 20 | - | 58526698 | 58526717 |
| *EIF2B5* | EIF2B5-RT-F | TCTGGGCTCTGGTACTGTCA | 34 | + | 20049679 | 20049698 |
|  | EIF2B5-RT-R | GACTGATGAATCTGTGCTCCA | 34 | - | 20050789 | 20050809 |

All positions are given for CanFam 2.0
